# Lost in traffic? The K^+^ channel of lily pollen, LilKT1, is detected at the endomembranes inside yeast cells, tobacco leaves, and lily pollen

**DOI:** 10.3389/fpls.2015.00047

**Published:** 2015-02-10

**Authors:** Minou J. Safiarian, Heidi Pertl-Obermeyer, Peter Lughofer, Rene Hude, Adam Bertl, Gerhard Obermeyer

**Affiliations:** ^1^Molecular Plant Biophysics and Biochemistry, Department of Molecular Biology, University of SalzburgSalzburg, Austria; ^2^Plant Systems Biology, University of HohenheimStuttgart, Germany; ^3^Yeast Membrane Biology, Department of Biology, Darmstadt University of TechnologyDarmstadt, Germany

**Keywords:** heterologous expression, K^+^ channel, *Lilium longiflorum*, pollen, tip growth, trafficking, yeast mutant

## Abstract

Fertilization in plants relies on fast growth of pollen tubes through the style tissue toward the ovules. This polarized growth depends on influx of ions and water to increase the tube’s volume. K^+^ inward rectifying channels were detected in many pollen species, with one identified in *Arabidopsis*. Here, an *Arabidopsis* AKT1-like channel (LilKT1) was identified from *Lilium longiflorum* pollen. Complementation of K^+^ uptake deficient yeast mutants was only successful when the entire LilKT1 C-terminus was replaced by the AKT1 C-terminus. No signals were observed in the plasma membrane (PM) of pollen tubes after expression of fluorescence-tagged LilKT1 nor were any LilKT1-derived peptides detectable in the pollen PM by mass spectrometry analysis. In contrast, fluorescent LilKT1 partly co-localized with the lily PM H^+^ ATPase LilHA2 in the PM of tobacco leaf cells, but exhibited a punctual fluorescence pattern and also sub-plasma membrane localization. Thus, incorporation of LilKT1 into the pollen PM seems tighter controlled than in other cells with still unknown trafficking signals in LilKT1’s C-terminus, resulting in channel densities below detection limits. This highly controlled incorporation might have physiological reasons: an uncontrolled number of K^+^ inward channels in the pollen PM will give an increased water influx due to the raising cytosolic K^+^ concentration, and finally, causing the tube to burst.

## INTRODUCTION

A pollen grain landing on the stigma surface generates a rapidly elongating pollen tube that is on target for the ovules which are often located at a far distance. Growth of pollen tubes is quite fast, e.g., 200–300 nm s^-1^ for *Lilium longiflorum* pollen *in vitro* cultures, and is restricted to the tube tip which is reflected by polar organization of organelles and localized growth-related cellular processes (Rosen et al., 1964; Feijo et al., 2004; Cole and Fowler, 2006; Cheung and Wu, 2007, 2008). For instance, secretory vesicles are transported by an actin cytoskeleton to the tube tip where they deliver new cell wall and membrane material whereas larger organelles (e.g., ER, Golgi, mitochondria) are excluded from this vesicle zone (Lancelle and Hepler, 1992; Foissner et al., 2002; Lovy-Wheeler et al., 2007; Kroeger et al., 2009). Furthermore, signal transduction pathways including reversible protein phosphorylation, phosphatidylinositol, G-proteins, cytosolic Ca^2+^ concentration and cytosolic pH form a regulatory network which controls tube growth.

Especially ion currents (Ca^2+^, H^+^, K^+^, Cl^-^) surrounding the growing pollen tube, have been identified as pacemakers of the growth rate and controllers of the direction of pollen tubes (Holdaway-Clarke and Hepler, 2003; Michard et al., 2009). Detailed studies showed an influx of Ca^2+^ into the tube tip (Holdaway-Clarke et al., 1997; Messerli et al., 1999) possibly mediated by glutamate receptor-like channels (Michard et al., 2011). The Ca^2+^ influx is restricted to the tip region and generates a tip-localized gradient of cytosolic Ca^2+^ which determines the tube’s growth direction (Obermeyer and Weisenseel, 1991; Malhó and Trewavas, 1996; Pierson et al., 1996; Michard et al., 2008; Iwano et al., 2009). At the pollen grain and partially along the tube shank, an active plasma membrane (PM) H^+^ ATPase transports H^+^ into the extracellular medium thus hyperpolarizing the PM and generating an outward current carried by H^+^ (Weisenseel and Jaffe, 1976; Obermeyer et al., 1992; Pertl et al., 2001; Certal et al., 2008) while chloride currents are detectable at the tube tip as eﬄuxes and at the tube shank as influxes (Zonia et al., 2002; Messerli et al., 2004). Ca^2+^-dependent anion channels are probably involved in the generation of these Cl^-^ currents (Tavares et al., 2011). Another major component of these currents are potassium ions (Weisenseel and Jaffe, 1976) which enter the pollen tube and leave at the tube tip (Michard et al., 2009). The uptake of K^+^ is important for tube growth by probably balancing the osmotic potential of the cytosol and the turgor pressure during rapid tube elongation (Benkert et al., 1997; Pertl et al., 2010; Winship et al., 2010; Zonia and Munnik, 2011). Ion channels permeable for K^+^ have been detected in intact lily pollen grains (Obermeyer and Blatt, 1995) and in protoplasts of *Arabidopsis*, *Brassica,* and *Lilium* pollen grains and tubes, respectively (Obermeyer and Kolb, 1993; Fan et al., 1999, 2001, 2003; Mouline et al., 2002; Griessner and Obermeyer, 2003; Becker et al., 2004). Generally, K^+^ influx was caused by voltage-gated and acidic pH-sensitive inward rectifying K^+^ channels that opened at membrane voltages more negative than –100 mV (Griessner and Obermeyer, 2003) but other K^+^ transporters might be involved in the generation of endogenous K^+^ inward currents, too. For instance, cyclic nucleotide-gated channels (cNGCs, Frietsch et al., 2007), cation/H^+^ exchangers (CHX, Sze et al., 2004), a tandem-pore K^+^ channel (AtTPK4, Becker et al., 2004) and non-specific cation channels (Wu et al., 2011) were detected in pollen. So far, SPIK (= AKT6, AT2G25600) is the only inward rectifying K^+^ channel that has been identified in pollen and that is responsible for the endogenous K^+^ currents (Mouline et al., 2002; Lebaudy et al., 2007). This K^+^ channel is exclusively expressed in pollen, exhibited strong inward rectifying properties and a knock-out mutant (*spik-1*) showed reduced inward K^+^ currents resulting in impaired pollen development and tube growth (Mouline et al., 2002) whereas the disruption of AtTPK4 had no effect on pollen development or tube growth (Becker et al., 2004). Therefore, SPIK is suggested as a major component responsible for K^+^ uptake during pollen tube growth.

Usually, four shaker-type subunits form a functional K^+^ channel (hetero- or homomeric) and each subunit consists of six transmembrane domains with a typical pore region amino acid sequence (GYGD) between transmembrane domains 5 and 6 (Dreyer et al., 1997; Long et al., 2005). Shaker-type channels were functionally analyzed by electrophysiological characterization of their transport properties in heterologous expression system like mammalian or insect cells (Gaymard et al., 1996), *Xenopus* oocytes (Schroeder, 1994), yeast cells (Bertl et al., 1997), or plant cells (Bei and Luan, 1998; Hosy et al., 2005). The first shaker-type K^+^ channels, AKT1 and KAT1, were identified in *Arabidopsis* and characterized in yeast mutants where they complemented the depleted, endogenous K^+^ uptake system (Anderson et al., 1992; Sentenac et al., 1992). Shaker-type K^+^ channels usually reside in the PM of plant cells to which they are delivered by secretory vesicles, e.g., during stomata opening (Hurst et al., 2004). The trafficking and incorporation of shaker-type K^+^ channels to the PM involves interactions with SNARE proteins (Sutter et al., 2006; Honsbein et al., 2009) and di-acidic motifs (e.g., DxE) in the C-terminus are crucial for their escape from the ER (Mikosch et al., 2006; Mikosch and Homann, 2009) as well as a C-linker domain behind the last transmembrane and the cyclic nucleotide-binding domain (Nieves-Cordones et al., 2014).

So far, the *Arabidopsis* shaker-type channels AKT1 and KAT1 are the best characterized plant K^+^ channels with important functions in root K^+^ uptake and control of the stomatal aperture. Despite the importance of K^+^ uptake during pollen tube elongation, SPIK/AKT6 is the only identified K^+^ channel subunit until now that may account for the detected inward K^+^ currents and the K^+^-dependent endogenous currents in pollen. To obtain a deeper insight in the behavior and the function of pollen K^+^ inward channels, we identified an AKT1-like K^+^ channel (LilKT1) from *L. longiflorum* pollen by *de novo* sequencing and detected the localization of the native as well as fluorescence-tagged and mutated LilKT1 proteins in pollen tubes, tobacco leaves and yeast cells.

## MATERIALS AND METHODS

### PLANT MATERIAL

*Lilium longiflorum* Thunb. plants (variety White Europe) were grown in the green house under environmental conditions with additional lighting (16 h day/8 h night). Mature pollen grains were harvested and used immediately or frozen in liquid nitrogen and stored at –80°C. Pollen grains were incubated in germination medium containing 10% (w/v) sucrose, 1.6 mM H_3_BO_3_, 1 mM KCl, 0.1 mM CaCl_2_, pH 5.6 adjusted if necessary with 2-[*N*-morpholin] ethanesulfonic acid (MES) or tris(hydroxymethyl) aminomethane (Tris).

Yeast mutant strains WΔ3 (*MAT*a *ade2 ura3 trp1 leu2 his3 trk1Δ::LEU2 trak2Δ::his3,* Haro et al., 1999) and PLY240 (*MAT*a *his*D*200 leu2-3, 112 trp1*D*901 ura3-52 suc2*D*9 trkD51 trk2*D*50::lox-kanMX-lox*) deleted for the Trk K^+^ uptake systems and the corresponding wild-type strains, W303 (*MAT*a *ade2 ura3 trp1 leu2 his3,* Wallis et al., 1989) and PLY232 (*MAT*a *his*Δ*200 leu2-3, 112 trp1*Δ*901 ura3-52 suc2*Δ*9*), respectively, were used for yeast complementation assays (Bertl et al., 2003). All yeast strains were grown in standard medium (YPD) containing 1% (w/v) yeast extract, 2% (w/v) peptone, and 2% (w/v) dextrose. For specific selection wild-type and transformed yeast strains were grown on minimal synthetic dextrose medium (SD, Clontech, Mountain View, CA, USA) with 0.67% (w/v) yeast nitrogen base without amino acids, 2% (w/v) glucose plus 2% (w/v) agar for solid media, and drop out (DO) supplement lacking specific components according to the corresponding selection marker on the incorporated plasmids.

### cDNA LIBRARY CONSTRUCTION AND SCREENING

A cDNA library was constructed with purified mRNA of *Lilium* pollen grains using the CloneMiner cDNA Library Construction Kit (Life Technologies, Vienna, Austria) according to the manufacturer’s instructions. During the cDNA synthesis, specific *att*B recombination sites were added to both ends of the cDNA inserts. These sites recombine with complementary *att*P sites present in the donor vector (pDONR222), which is then incorporated into DH10B T1 phage resistant *Escherichia coli* cells. Total RNA was isolated according to Chang et al. (1993) or RNAeasy Plant Mini Kit (Qiagen, Hilden, Germany) and mRNAs were isolated from total RNA using mRNA Mini Kit (Qiagen). The cDNA library (5 × 10^5^ cfu) was screened with a digoxigenin-labeled K^+^ channel probe which was obtained by PCR using an AmpliTaq DNA-polymerase (Applied Biosystems, Vienna, Austria), degenerated primer pair H5_fwd and S6_rev (Table S1) and a digoxigenin-labeling mix (Roche Diagnostics, Vienna, Austria) with the following amplification conditions: 3 min at 94°C, 35 cycles (40 s 94°C, 40 s 48°C, and 1 min 72°C), 7 min 72°C following the manufacturer’s instructions. DNA of the grown colonies was transferred and cross-linked to Hybond N^+^ Nylon membranes (GE Healthcare, Vienna, Austria) which were hybridized with the DIG-labeled K^+^ channel probe using standard molecular biology techniques (Sambrook and Russell, 2001). Washing with SSC buffer (20 × SSC buffer: 3 M NaCl, 0.3 M Na citrate, pH 7.0 adjusted with NaOH) of increasing stringency from 0.1 × SSC plus 0.1% (w/v) SDS to 2 × SSC plus 0.2% (w/v) SDS at 62°C removes unspecific bound probes. The bound DIG-labeled probe was detected with an anti-DIG antibody conjugated with alkaline phosphatase (Sigma, Vienna, Austria) in maleate buffer (100 mM maleic acid, 150 mM NaCl, pH 7.5 adjusted with NaOH). Positive clones were isolated and checked by PCR using the H5_fwd and S6_rev primer pair.

### RACE- AND SOE-PCR

To obtain the missing 5′- and 3′-end of the positive clones, a RACE (rapid amplification of cDNA ends)-PCR was performed using the SMART (Switching Mechanism at 5′ end of RNA Transcript) RACE method according to the manufacturer’s instructions (BD Biosciences Clontech, Palo Alto, CA, USA). Insert-specific primer GYGDLHA_rev and H5spec_fwd (Table S1) together with the supplied 5′-end and 3′-end primer for 5′-RACE and 3′-RACE, respectively, were used. RACE–PCR products were cloned into TOPO-TA vectors (Life Technologies) and checked for inserts by PCR using vector backbone primer pair combinations (M13_fwd and M13_rev) as well as insert-specific primer pairs (H5spec_fwd, H5spec_rev, GYGDLHA_rev, Table S1).

Splicing by overlap extension (SOE)-PCR was performed to combine the two RACE–PCR products which overlapped in the sequence between the primer H5spec_fwd and GYGDLHA_rev. The 5′-end part of LilKT1 was amplified by AmpliTaq DNA polymerase using the primer LilKT1_fwd and GYGDHLA_rev and the following PCR conditions: 95°C 10 min, 30 cycles (95°C 30 s, 55°C 30 s, 72°C 1 min), 72°C 5 min. The 3′-end part of LilKT1 was amplified by AmpliTaq DNA polymerase using the primer LilKT1_rev and H5spec-fwd and the following PCR conditions: 94°C 10 min, 30 cycles (94°C 30 s, 55°C 30 s, 72°C 4.5 min), 72°C 7 min. Both PCR products were cut out from 1% (w/v) agarose gels and purified using the Ultrafree-DNA kit (Millipore, Vienna, Austria). To combine both parts, the 5′- and 3′-ends were used as templates in a PCR experiment with LilKT1_fwd and LilKT1_rev as specific primer and the full-length sequence of LilKT1 was amplified using AmpliTaq DNA polymerase with the following PCR conditions: 95°C 10 min, 30 cycles (95°C 30 s, 53°C 30 s, 72°C 3 min), 72°C 10 min. The resulting product was cloned into TOPO-TA or pENTR (Life Technologies) vectors. The insert was verified by control PCRs and sequenced (Eurofins/MWG, Ebersberg, Germany). All standard molecular biology methods were performed according to Sambrook and Russell (2001).

### CONSTRUCTION OF YEAST, POLLEN, AND PLANT EXPRESSION VECTORS

For yeast expression, the sequence of LilKT1 (accession number EF397611) was chemically synthesized giving yLilKT1 (Sloning BioTechnology, Puchheim, Germany) and cloned into pCR plasmids with the following sequence modifications: codon usage was optimized for yeast expression and two recombination sites, *rec1* and *rec2*, flanking the yLilKT1 sequence allowing homologous recombination cloning with pGREG plasmids (Jansen et al., 2005). Cloning yLilKT1 into pGREG535 resulted in galactose-inducible, N-terminal HA (hem agglutinin) tagged LilKT1 protein. Cloning was performed by co-transformation of yeast cells with a *Sal*I linearized pGREG535 and the yLilKT sequence flanked by *rec1* and *rec2* sequences. The *Arabidopsis thaliana* pollen-specific K^+^ channel, AKT6, was amplified from the plasmid pCI-AKT6 with specific primer plus overhangs for the *rec1* and *rec2* sides, respectively (Table S1). The resulting PCR product was also cloned into the yeast expression vector pGREG535.

For expression in tobacco plants, two binary vectors (pK7YLilKT1.2 and pK7CLilHA2.2) were constructed using the plasmids pK7YWG2 and pK7CWG2 (Karimi et al., 2002, 2007^1^) containing a kanamycin resistance gene under the control of the NOS-promoter, a gyrase inhibitor gene (cddB) flanked by *att*R1 and *att*R2 sites. The LilKT1 and the lily pollen PM H^+^ ATPase LilHA2 (accession number EF397610.2) were cloned into the binary vectors via LR recombination (LR clonase reaction mix, Life Technologies). The inserted genes were expressed with N-terminal fusions of fluorescent proteins: eCFP fused to LilHA2 and eYFP fused to LilKT1.

For expression in lily pollen, the plasmid pLAT52-pA2 (Sommer et al., 2008), which consists of the pBS-KS(+) vector backbone containing the pollen-specific promoter LAT52 (Twell et al., 1990), a multiple cloning site and a poly adenylation site, was modified as follows: restriction sites not present in the LilKT1 sequence (*Avr*II, *Aat*II, and *Xma*I) were added by inserting the two assembled oligonucleotides (5′-catggcctagggctgacgt cgctcccgggctgca-3′ and 3′-cggatcccgactgcagcgagggccc-5′) between the original *Nco*I and *Pst*I restriction sites. To obtain a C-terminal YFP fusion to LilKT1, the restriction sites *Avr*II/*Aat*II and *Aat*II/*Xma*I were added by PCR to the LilKT1 and YFP sequences, respectively. The plasmid pLAT52-pA2 was first linearized by digestion with *Avr*II and *Aat*II and LilKT1 (without its original stop codon) was inserted. In a second step, pLAT52-pA2-LilKT1 was digested with *Aat*II and *Xma*I and the YFP sequence with the additional restriction sites was inserted, giving pLAT52-YFP::LilKT1. An N-terminal YFP fusion to LilKT1 was constructed in a similar way and named pLAT52-LilKT1::YFP. Alternatively, the CaMV promoter of the GATEWAY-compatible plasmids p2YGW and p2GWY (Karimi et al., 2007) was replaced by the LAT52 or ZM13 promoter (Hamilton et al., 1992) using the *Sac*I and *Spe*I restriction sites, thus giving N-terminal and C-terminal fusions of YFP to LilKT1 under the control of a pollen-specific promoter.

### GENERATION OF MUTATIONS IN yLilKT1

Additional di-acidic motifs were inserted in yLilKT1 by PCR-based site-directed mutagenesis (round-the-horn^2^) and confirmed by sequencing. A complementary primer pair containing the mutation was used to create a linearized product of the entire plasmid. The primers were phosphorylated with T4 PNK (Fermentas) to allow self-ligation of the PCR product forming a circular plasmid again which was incorporated into DH5α *E. coli*. Mutagenesis was performed with pCR containing yLilKT1 and the appropriate *rec* sites for cloning with the yeast expression vector pGREG535. The amplifications were performed using the Phusion Polymerase (Biozym, Vienna, Austria) with the complementary primer pairs G797D_fwd/rev and K840D_fwd/rev (Table S1) and the following PCR conditions: 98°C 30 s, 30 cycles (98°C 10 s, 66°C 30 s, 72°C 8 min), 72°C 15 min.

For construction of the chimera channel LilKT1/AKT1, both fragments were produced by separate PCRs using Phusion polymerase. The yLilKT1-N-terminus was amplified from pGREG535 containing the entire yLilKT1 sequence using a forward primer SOE_N_LilKT1_*rec1*_fwd and a reverse primer SOE_N_LilKT1_rev (Table S1). The amplification was performed using AmpliTaq polymerase (Applied Biosystems): 98°C 30 s, 30 cycles (98°C 10 s, 66°C 30 s, 72°C 1 min), 72°C 10 min giving a PCR product of exactly 1 kb. The C-terminus of AKT1 was amplified from the pFL61-AKT1 plasmid with a forward primer SOE-AKT1-C containing a part of the *rec2*-side as an overhang (98°C 30 s, 30 cycles (98°C 10 s, 68°C 30 s, 72°C 2 min), 72°C 10 min), giving a PCR product of 1,626 kb. Both PCR products were gel-purified, extracted and fused by SOE-PCR using an overlapping sequence of about 14 bp at their end and start, respectively: 10 pre-cycles were performed without the addition of primer (98°C 30 s, 10 cycles (98°C 10 s, 45°C 30 s, 72°C 3 min), 72°C 10 min). Afterward, primer for adding *rec1* and *rec2* sides to the entire product were added. The fused product was amplified by AmpliTaq Polymerase to produce A-overhangs for direct cloning into pGEM-T Easy (Promega). In addition, a truncated yLilKT1 lacking its C-terminus was produced by adding the *rec2*-side to the Lil-N-fragment by PCR. The resulting fusion protein (chim_LilKT1) as well as the truncated yLilKT1 (Δ323-862, ΔCterm_LilKT1) were cloned into the pGREG535 vector (Figure S4).

### EXPRESSION OF LilKT1 IN YEAST MUTANTS AND FUNCTIONAL COMPLEMENTATION

The yeast wild-type and mutant strains were transformed by the Li^+^ acetate method (Gietz and Schiestl, 2007) with plasmids pGREG535-yLilKT1, pFL61-AKT1 (Minet et al., 1992; Sentenac et al., 1992), pYES2-KAT1 (Nakamura et al., 1997) and empty plasmids pYES2 and pGREG535 for control experiments. Double transformations were generated by incorporating the URA3 carrying plasmids pFL61-AKT1 or pYES2-KAT1 into yeast cells already containing the LEU2 carrying plasmid pGREG-yLilKT1. Double transformed yeast cells were selected on SD medium lacking uracil and leucine (SD/-ura/-leu). For a qualitative assessment of growth phenotypes, cells were grown for 18 h at 30°C in SD/-ura/-leu supplemented with 100 mM KCl. Cells were harvested by centrifugation of 1 ml samples at 4,000 rpm and resuspended in sterile, double-distilled water to an optical density of OD_600_ = 1.0 ± 0.05 corresponding to about 2 × 10^7^ cells ml^-1^. Serial tenfold dilutions were prepared and 10 μl aliquots of each dilution were spotted on SGal/Raf-agar plates containing varying KCl concentrations. The plates were incubated at 30°C for 3 days and yeast growth was monitored by imaging the agar plates at 600 dpi resolution. Growth curves were obtained from yeast double transformants that initially grew on SD/-ura/-leu agar plates containing 100 mM KCl. Single clones were transferred to 5 ml culture medium (SD/-ura/-leu) and incubated for 24 h. Cells were harvested by centrifugation of 1 ml aliquots, washed twice with sterile water and transferred to 10 ml SGal/Raf -ura-leu containing 100 mM KCl with an initial cell density corresponding to OD_600_ = 0.1 ± 0.02. Cell growth was monitored at 600 nm (U-1800, Hitachi, Tokyo, Japan). The complementation assays with LilKT1 mutants and AKT6 were performed similarly.

### YEAST AND LILY POLLEN ORGANELLE VESICLE PREPARATION

Yeast organelles were prepared according to Villalba et al. (1992). Yeast cells (0.5–1 l culture volume) were pelleted by centrifugation (5,000x*g*, 10 min, RT) and resuspended in membrane breaking buffer (0.4 M saccharose, 1 mM EDTA, 10 mM Tris/HCl pH 7.4) supplemented with protease inhibitor cocktail (Roche Diagnostics, Vienna, Austria). Acid-washed glass beads (425–600 μm diameters, Sigma) were added to a level slightly below the meniscus. Cells were disrupted by vigorous vortexing for 15 min with 15 s intervals of vortexing followed by 15 s cooling on ice. The lysate was collected and centrifuged at 1,000x*g* for 15 min at 4°C to remove cell debris. The supernatant (homogenate) was centrifuged at 113,000x*g* for 1 h at 4°C (Sorvall Discovery 100SE, Kendro, Langenselbold, Germany). The supernatant [soluble, cytosolic fraction (CF)] was collected and the pellet [membrane fraction (MF)] was resuspended in membrane buffer (10 mM Tris/HCl pH 7.4, 1 mM EDTA, 1 mM DTT). The MF (300 μl) was loaded on the top of a continuous sucrose gradient (15 ml) ranging from 20 to 53% (w/w) sucrose in membrane buffer with a 60% (w/w) sucrose cushion at the bottom. The gradient was centrifuged in a swing-out rotor (AH629, 17 ml, Sorvall) at 108,000x*g* for 16 h at 4°C. Fractions of 0.5 ml were collected from bottom of the pierced centrifugation tube by applying slight pressure on the top of the gradient. Protein concentrations and sucrose densities were measured in each fraction using a DC protein assay (Biorad, Vienna, Austria) and a refractometer (Optotech, Munich, Germany). The sucrose concentration of all fractions was diluted to 20% (w/v) and fractions were pelleted at 120,000 g for 1 h at 4°C in a fixed-angle rotor (TFT45.6, Sorvall). Pellets were resuspended in 100 μl membrane buffer.

Organelles from lily pollen incubated for 0, 10, 30, 60, and 240 min in germination medium were separated on a discontinuous sucrose gradient as described by Pertl et al. (2005, 2009).

### GEL ELECTROPHORESIS AND IMMUNODETECTION

Sodium dodecyl sulfate gel electrophoresis (mini PROTEAN III system, Biorad) was performed according to Laemmli (1970) as described in Pertl et al. (2005). Proteins were either stained with Coomassie brilliant blue (CBB) or transferred to nitrocellulose (NC) membranes using a semi-dry blotting system (Biorad). For immunodetection, NC membranes were blocked with 3% (w/v) casein in PBS (phosphate-buffered saline)-Tween overnight at 4°C, washed 3 × 5 min in PBS-Tween, incubated with primary antibody for 1 h in PBS-Tween, washed 3 × 5 min, incubated with secondary antibody for 1 h, washed for 3 × 5 min in PBS-Tween. Alkaline phosphatase activity (AP) was detected using 0.175 mg ml^-1^ 5-bromo-4-chloro-3-indolyl-phosphate *p*-toluidine salt (BCIP) and 0.3375 mg ml^-1^ nitroblue tetrazolium chloride (NBT) in detection buffer (100 mM Tris/HCl, pH 9.5, 100 mM NaCl, 50 mM MgCl_2_). Horse radish peroxidase activity (HRP) was detected by enhanced chemi-luminescence (ECL, GE Healthcare). The following antibody combinations were used: anti-yeast PM H^+^ ATPase (PMA1, 1:1,000, gift from Ramon Serrano) and monoclonal anti-rabbit IgG AP-conjugated (1:8,000, Sigma) or monoclonal anti-rabbit IgG HRP-conjugated (1:20,000, Sigma); anti-dolichol phosphate mannose synthase (1:2,000, Life Technologies, Vienna, Austria) and anti-mouse IgG AP-conjugated (1:10,000, Sigma); anti-yeast V0-ATPase subunit (1:1,000, Life Technologies) and anti-mouse HRP-conjugated (1:10,000, Sigma); anti-yeast V1-ATPase subunit (1:1,000, Life Technologies) and anti-mouse HRP-conjugated (1:10,000, Sigma); anti-HA-tag (1:10,000, Abcam, Cambridge, UK) and anti-mouse IgG HRP-conjugated (1:10,000, Sigma); anti-AKT1 (1:250, Agrisera, Vännäs, Sweden) and monoclonal anti-rabbit IgG HRP-conjugated (1:10,000, Sigma).

### MASS SPECTROMETRY AND ANALYSIS

Proteins of lily pollen organelle fractions prepared by discontinuous sucrose gradient centrifugation were separated by SDS-PAGE and stained with CBB. Each gel lane was cut into small pieces (1 × 1 mm) and subjected to *in-gel* trypsin digestion (Olsen et al., 2004). All peptide mixtures were analyzed by LC-MS/MS (Niittylä et al., 2007) and fragmentation spectra were searched against a custom-made protein data base containing only lily pollen membrane proteins including LilKT1 according to Pertl et al. (2009).

### TRANSFORMATION OF LILY POLLEN AND TOBACCO LEAVES

Pollen grains were transformed by particle bombardment according to Sommer et al. (2008). Gold beads (1.6 μm diameter) were coated with the pLAT52-YFP::LilKT1 or the pLAT52-LilKT1::YFP plasmids and cartridges were prepared according to the supplier’s instructions (Biorad). Lily pollen grains of 1 mature anther were resuspended in medium [in mM: 680 mannitol, 5 CaCl_2_, 10 KCl, 0.5 ascorbic acid, 10 MES adjusted with 1,3-bis(tris(hydroxymethyl)methyl-amino)propane (BTP) to pH 6.0], placed on a filter paper, bombarded twice with a Helios Gene Gun (Biorad, pressure = 200 psi) and incubated at room temperature for at least 24 h in the dark. Bombarded pollen grains were carefully transferred to germination medium and inspected every 20 min for germination and fluorescence.

Tobacco leaves (*Nicotiana tabacum*) were transiently transformed as described in Obermeyer et al. (2004).

### CONFOCAL LASER-SCANNING MICROSCOPY

Fluorescent pollen grains were observed with a LSM 510 (Zeiss, Oberkochen, Germany) using the argon laser for excitation (488 nm) and emission was monitored through a band pass filter (505–550 nm). In tobacco and yeast cells, fluorescent proteins were localized using Leica DMI4000 B with HCX PL APO lambda blue 63x/1.2 water UV objective and standard laser and filter settings for CFP and YFP detection.

## RESULTS

**FIGURE 1:**
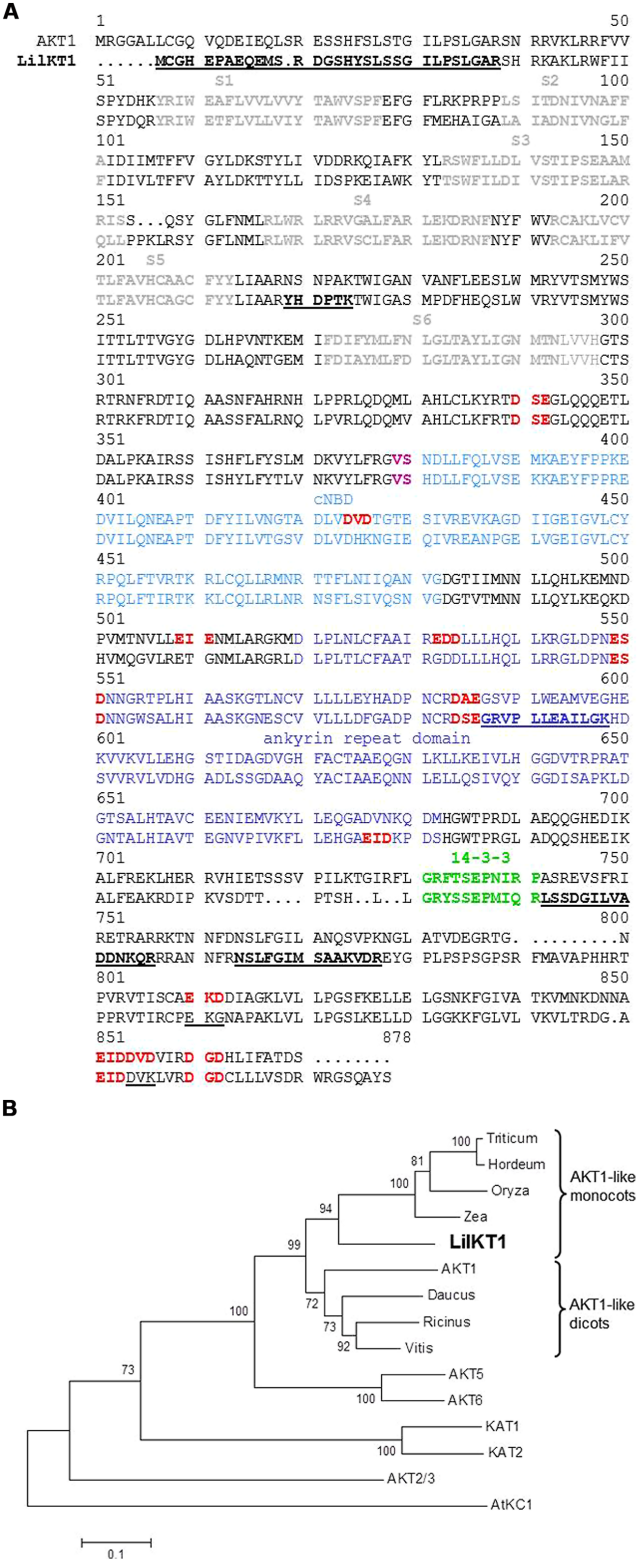
**Sequence alignment of LilKT1 with plant inward-rectifying K^+^ channels. (A)** Alignment of LilKT1 with AKT1 channel subunit from *Arabidopsis thaliana* showing the typical Shaker-like channel motifs and domains: transmembrane domains S1–S6 (bold, gray), binding domain for cyclic nucleotides (cNBD, blue), V and S (magenta) are important residues in the C-linker domain, ankyrin repeat domain (dark blue), putative 14-3-3 binding domain (green). Peptides underlined and bold were identified by LC-MS/MS. Di-acidic motifs at the C-terminus were indicated as red and bold. Mutated di-acidic motifs are underlined. **(B)** Phylogenetic tree of well-known shaker-like plant K^+^ channel subunits and the first eight protein sequences with the highest homology to LilKT1 (BLAST search, see Table S2 for accession numbers).

A new K^+^ channel with high homology to the *Arabidopsis* inward K^+^ rectifier AKT1, was identified in lily pollen using a combination of cDNA library screening, RT-PCR and RACE–PCR (**Figure 1**). In a first attempt, a lily pollen cDNA library (5 × 10^5^ clones) was screened with a digoxigenin-labeled, K^+^ channel-specific probe that was generated by RT-PCR using the degenerated primer pair H5_fwd and S6_rev (Table S1). With this approach, no K^+^ channels could be detected, although two PM H^+^ ATPases were identified in a parallel screen of the same cDNA library.

In a second approach, a reverse transcription reaction was performed with S6_rev as a specific reverse primer. From the resulting cDNA, a 215 bp long product was amplified by PCR with the plant K^+^ channel specific, but degenerated primer pair H5_fwd and S6_rev (Table S1). The missing 5′- and 3′-ends of the partial K^+^ channel sequence were obtained by RACE–PCR resulting in two partial sequences (5′-end to S6_rev and H5_fwd to 3′-end) which were finally assembled to one full-length sequence by SOE-PCR. The final product showed a high homology to the AKT1-like K^+^ channel subunits (Table S2 and **Figure 1A**) and thus, was named LilKT1 (accession number EF397611). A phylogenetic tree which was generated using protein sequences showing the highest homology to LilKT1 and the well-known *Arabidopsis* K^+^ channels (**Figure 1B**), revealed a close relation of LilKT1 to other monocot AKT1-like channels. LilKT1 showed the typical topology and domains of plant inward rectifying channels: six transmembrane domains and the K^+^ channel-specific amino acid sequence GYGD between the transmembrane domains S5 and S6. A binding domain for cyclic nucleotides (cNBD) and the ankyrin repeat domain classified LilKT1 as a member of the AKT1-like channel group (**Figure 1A**).

### HETEROLOGOUS EXPRESSION OF LilKT1 IN YEAST

**FIGURE 2:**
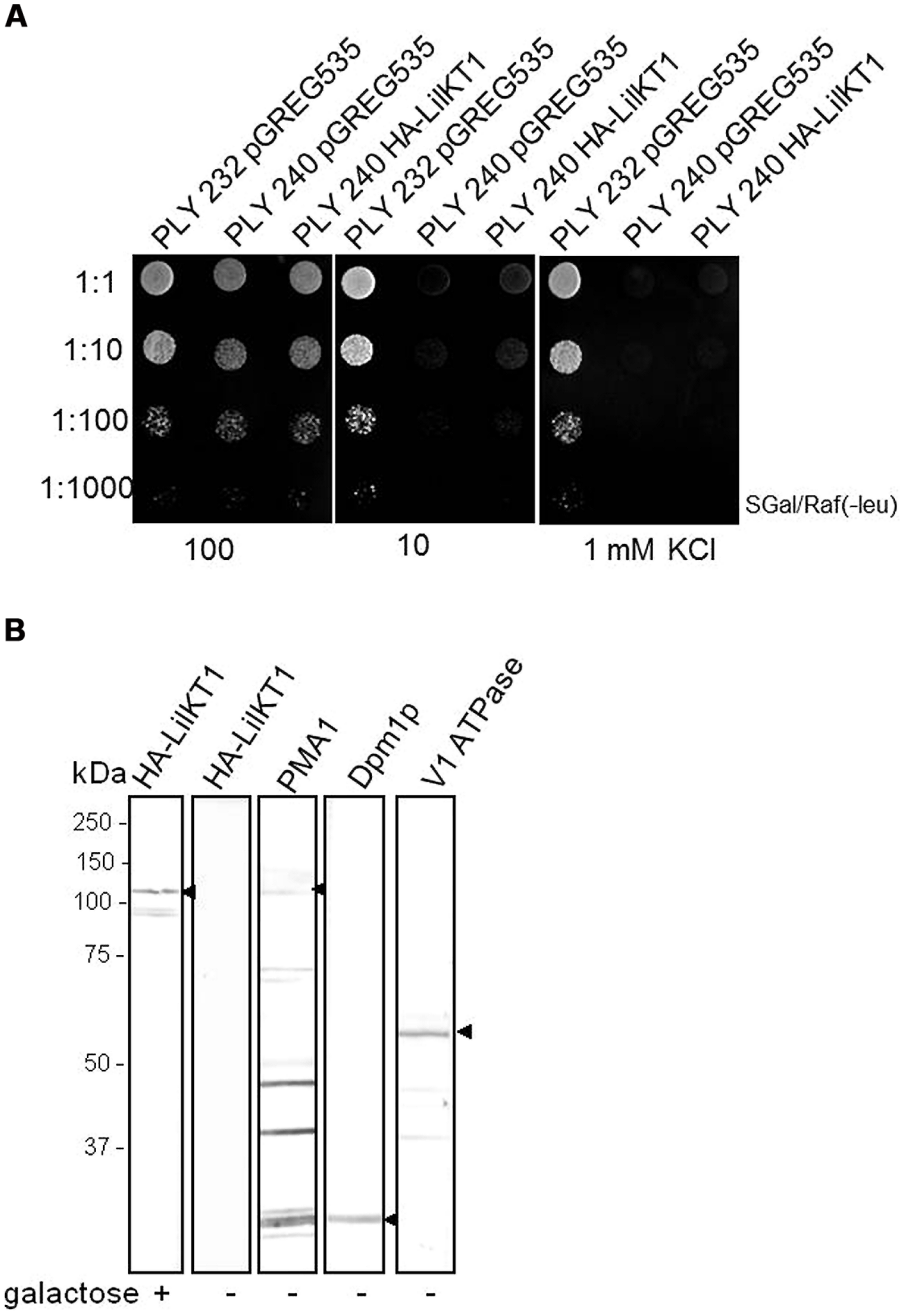
**Functional complementation assay and expression of LilKT1 in yeast K^+^ uptake mutant. (A)** PLY240 mutants expressing HA-yLilKT1 could not grow at low K^+^ concentrations. Control experiments were performed with the wild-type strain PLY232 containing the empty expression vector pGREG535. **(B)** HA-tagged LilKT1 was detected in the membrane fraction of PLY 240 after induction with galacatose (+). The respective organelle membrane markers, Pma1p (plasma membrane H^+^ ATPase), Dpm1p (dolichol phosphate mannose synthase of ER) and the Vma1p subunit of the vacuolar V-type ATPase (V1-subunit) were detected in non-induced yeast cells. Arrowheads mark the expected molecular weights. Additional protein signals in the Pma1p lane are caused by secondary antibody. 40 μg protein per lane.

To test whether LilKT1 can form functional K^+^ channels and to characterize the transport properties, LilKT1 was expressed in yeast mutant strains WΔ3 and PLY240 which lack the endogenous K^+^ uptake system. In accordance with previous plant K^+^ channel characterization studies with the closely related AKT1 protein, LilKT1 would be able to complement for the lacking endogenous K^+^ transport system. A chemically synthesized LilKT1 sequence with optimized codon usage for yeast expression (yLilKT1) was expressed with an N-terminal HA-tag to monitor the expression of LilKT1 (**Figure 2**). A functional complementation assay using the K^+^ uptake mutants PLY240 showed that yeast mutants expressing LilKT1 could not grow at KCl concentrations ≤10 mM and therefore, were unable to restore the function of the removed yeast K^+^ transporters (**Figure 2A**). A similar result was obtained with the K^+^ uptake mutant WΔ3 (Figure S2) whereas in control experiments performed in parallel, the well-characterized plant K^+^ channels AKT1 and KAT1 were able to reconstitute the lacking endogenous K^+^ uptake system of PLY240 (Figure S3). The yeast mutants containing AKT1 or KAT1 in the respective expression vector were able to grow almost as well in media with low KCl concentrations (10 or 1 mM) as the corresponding wild-type strain PLY232, while no growth was observed in PLY240 cell transformed with the empty vector. Upon induction with galactose, the expression of LilKT1 in PLY240 mutants was detected with HA-specific antibodies (**Figure 2B**): a single protein band in the range of the expected molecular weight of 7xHA::LilKT1 (107 kDa) was recognized in the crude MF of yeast strain PLY240 whereas no signals were observed in the MF of non-induced cells (**Figure 2B**, 2nd lane). In addition, marker proteins specific for yeast organelles, were detected and gave a single signal for the ER-localized dolichol phosphate mannose synthase (Dpm1p, ca. 30 kDa, **Figure 2B**, lane 4), specific signals for the vacuolar H^+^ ATPase subunits Vma1p (ca. 60 kDa, lane 5) and a signal at the expected size of 100 kDa for the PM H^+^ ATPase (Pma1p, lane 3). The signal for the Pma1p was quite weak and additional bands were caused by unspecific reactivity of the secondary antibody. However, other combinations of primary and secondary antibodies for recognition of the Pma1p were tested, e.g., commercial available anti-Pma1p antibody (Abcam, Cambridge, UK) but without improving the specificity (data not shown). Although LilKT1 protein is expressed in yeast, it could not complement the yeast K^+^ uptake mutants, and may therefore not be localized in the PM, which is essential for reconstitution of the K^+^ uptake.

**FIGURE 3:**
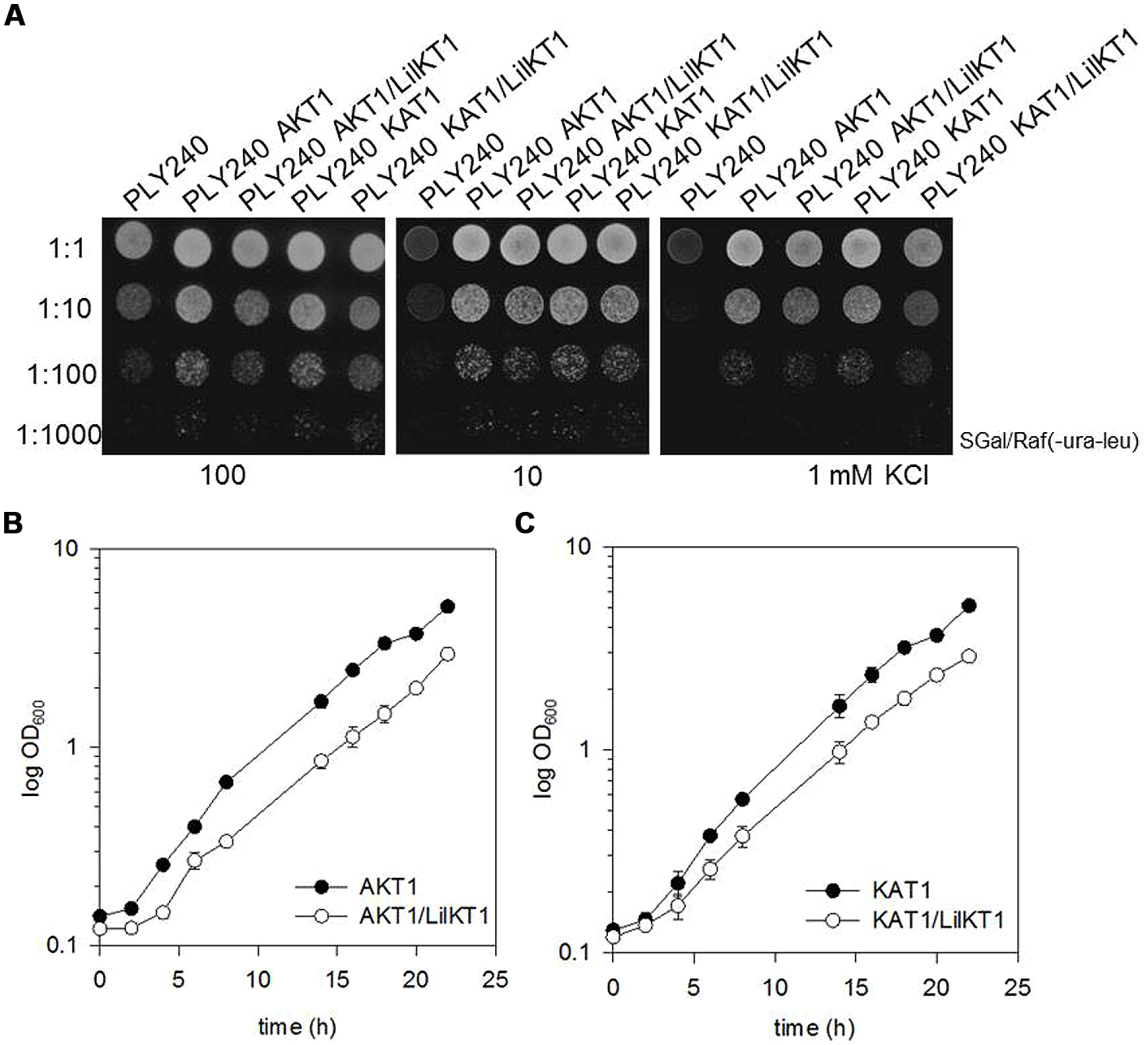
**Co-expression of LilKT1 with AKT1 and KAT1. (A)** Drop assay with K^+^ uptake deficient yeast strain PLY240 complemented with AKT1, KAT1 as well as with the combinations LilKT1/AKT1 and LilKT1/KAT1. **(B)** Growth curves of the transformed PLY240 yeast cells show slower growth rates of double-transformed (AKT1/LilKT1) compared to single-transformed (AKT1) yeast. **(C)** Growth of PLY240 mutants transformed with KAT1, or KAT1/LilKT1 *n* = 3 ± S.D. Channels were cloned in their respective plasmids: LilKT1 in pGREG535, KAT1 in pYES and AKT1 in pFL61. Medium in **(B,C)** contained 100 mM KCl.

To test whether formation of heterotetrameric channels may direct LilKT1 subunits to the PM, the yeast mutant PLY240 was co-transformed with pGREG535-HA::LilKT1 and pFL61-AKT1. Double transformants did express both channel proteins as verified by immunodetection with antibodies against the AKT1 protein and the HA-tag of LilKT1 using a MF prepared from double transformants grown on galactose medium (data not shown). As expected, co-transformation of LilKT1 with AKT1 or KAT1 could reconstitute the K^+^ uptake defect in PLY240 shown by the ability of double transformants (PLY240 AKT1/LilKT1 and PLY240 KAT1/LilKT1) to grow on low K^+^ media (**Figure 3**). However, co-expression of AKT1 and KAT1 with LilKT1 inhibited yeast growth slightly resulting in less dense growth (**Figure 3A**, best visible at dilution 1:10). To verify this observation single (AKT1) and double-transformed yeast mutants (AKT1/LilKT1) were grown in liquid media in the presence of 100 mM KCl. Compared to the growth of AKT1- and KAT1-expressing yeast cells the double transformants (AKT1/LilKT1 and KAT1/LilKT1) grew significantly slower (**Figures 3B,C**) indicating that AKT1 or KAT1 are less able to complement the K^+^ uptake mutation in the presence of LilKT1.

**FIGURE 4:**
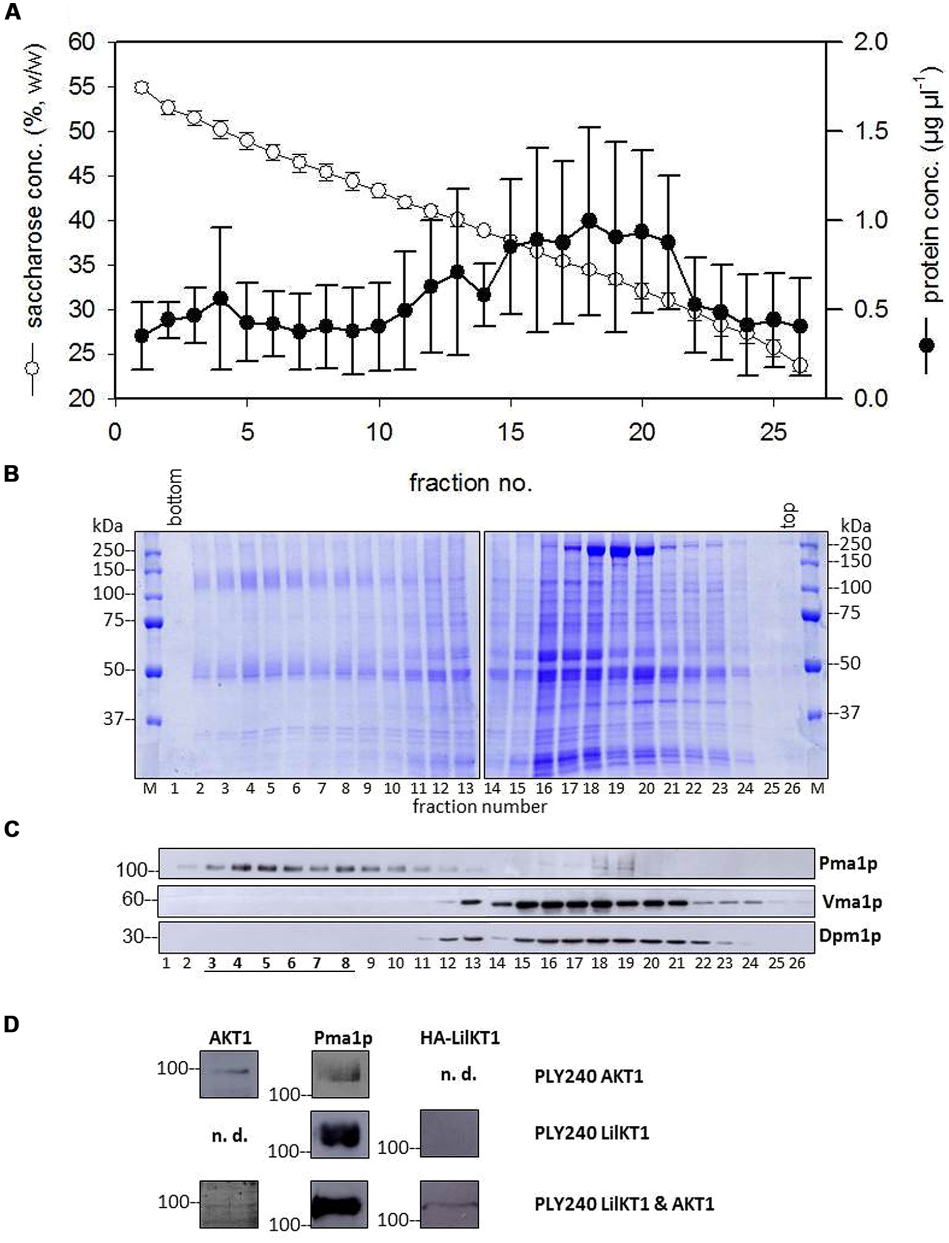
**Co-sedimentation of LilKT1 with yeast organelle markers. **(A)**** Distribution of membrane protein and sucrose concentration in a continuous sucrose gradient collected after 16 h centrifugation (*n* = 5 ± S.D.) **(B)** Typical pattern of membrane proteins along the sucrose gradient. 30 μl of sucrose-adjusted fraction loaded per lane. **(C)** Typical distribution of plasma membrane (Pma1p), ER (Dpm1p), and vacuole (Vma1p) marker enzymes in the sucrose gradient. **(D)** Plasma membrane fractions (3–8) of three gradients were pooled, proteins were precipitated and transferred to NC membranes after SDS-PAGE. The proteins AKT1, Pma1p and the HA-tag of LilKT1 were detected with respective antibodies in the pooled fractions of PM fractions (underlined) of PLY240 yeast mutants expressing AKT1 or LilKT1 alone and the combination of AKT1 and LilKT1 (n.d., not determined).

To investigate whether AKT1 can assist the incorporation of LilKT1 into the PM, a co-sedimentation analysis was performed (**Figure 4**). The majority of proteins sedimented at lower sucrose densities between fractions 15 and 21 (**Figures 4A,B**). In this density range the proteins Dpm1p and Vma1p marking ER and vacuolar membranes, respectively, were detected (**Figure 4C**) whereas the PM marker Pma1p was observed at higher densities (fractions 3–8) allowing the separation of PM from endomembranes (**Figure 4C**). In yeast cells expressing AKT1 (PLY240 pFL61-AKT1) the AKT1 protein co-sedimented with the PM H^+^ ATPase Pma1p (**Figure 4D**, upper row) although the immunodetection signal caused by AKT1 was only visible when fractions 3–8 of three sucrose gradients were pooled, precipitated and analyzed. Using the same procedure for yeast cells expressing HA::LilKT1 only, no signals were visible for HA-tagged LilKT1, while Pmap1 was detectable in the pooled fractions (**Figure 4D**, middle row). When HA::LilKT1 was co-expressed with AKT1 both channel proteins were detectable together with Pma1p in the pooled and concentrated PM fraction (**Figure 4D**, lower row). Note that in PM fractions from yeast cells co-expressing both channel subunits, the AKT1 signal was weaker than in preparations of yeast cells that expressed AKT1 only.

**FIGURE 5:**
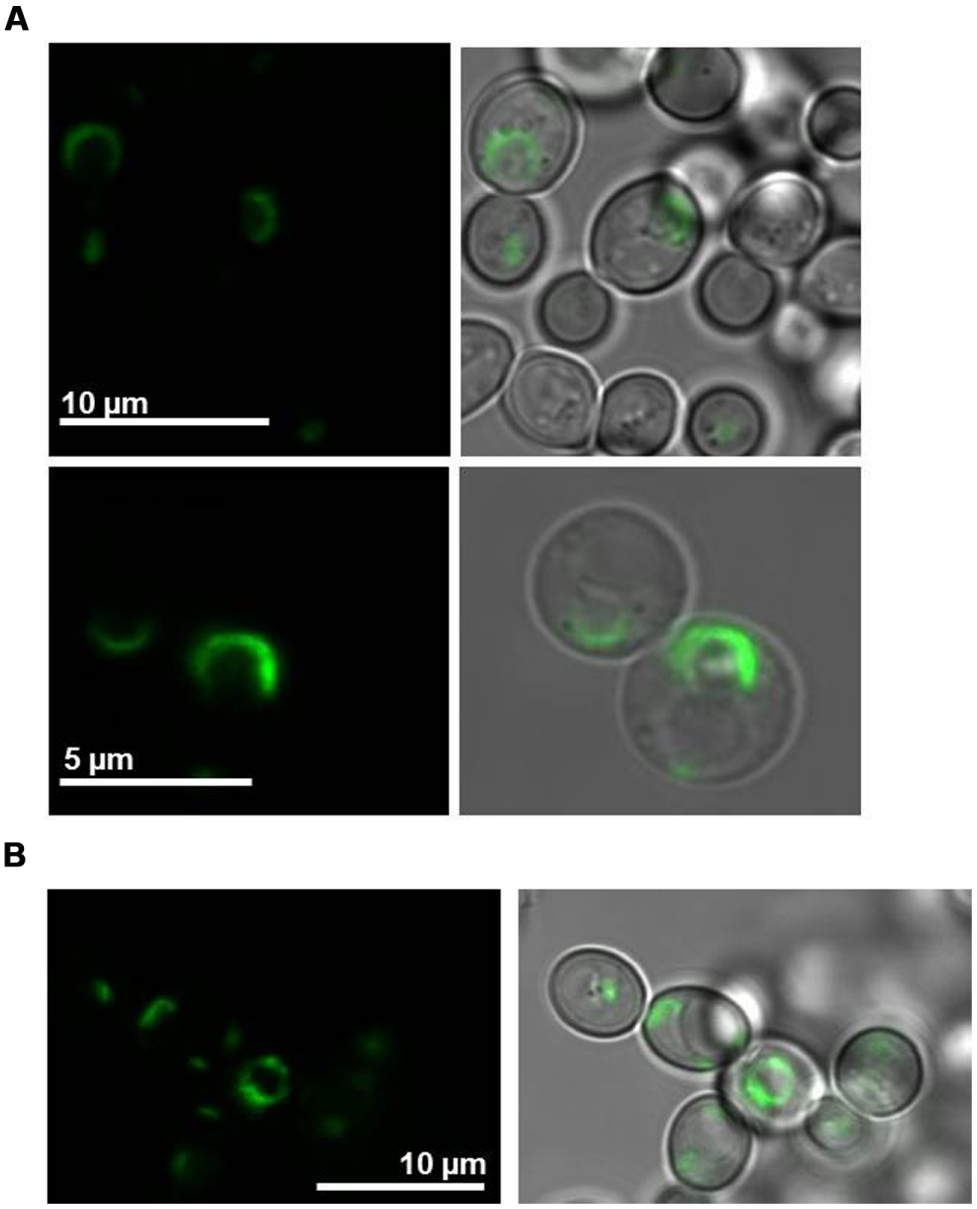
**Localization of fluorescence-tagged LilKT1 in yeast protoplasts. (A)** N-terminally GFP fused LilKT1 was expressed in yeast cells and localizes in structures surrounding the nucleus (ER). Upper panel: yeast cells, lower panel: yeast cell protoplasts. Fluorescence images are on the left and bright field images merged with fluorescence images on the right.** (B)** Localization of GFP::LilKT1 co-expressed with AKT1 in yeast cells. Co-expression with AKT1 shows the localization in similar compartments as expression of LilKT1 alone **(A)**.

The co-sedimentation assay predicted localization of LilKT1 at the PM in the presence of AKT1. Expression of GFP::LilKT1 alone showed fluorescence in a compartment around the nucleus which is very likely ER (**Figure 5A**). However, during co-expression of GFP::LilKT1 with AKT1 (**Figure 5B**), only fluorescence signals in the ER but not in the PM were detectable indicating a quantity of LilKT1 proteins in the PM below the detection limit of fluorescence microscopy.

**FIGURE 6:**
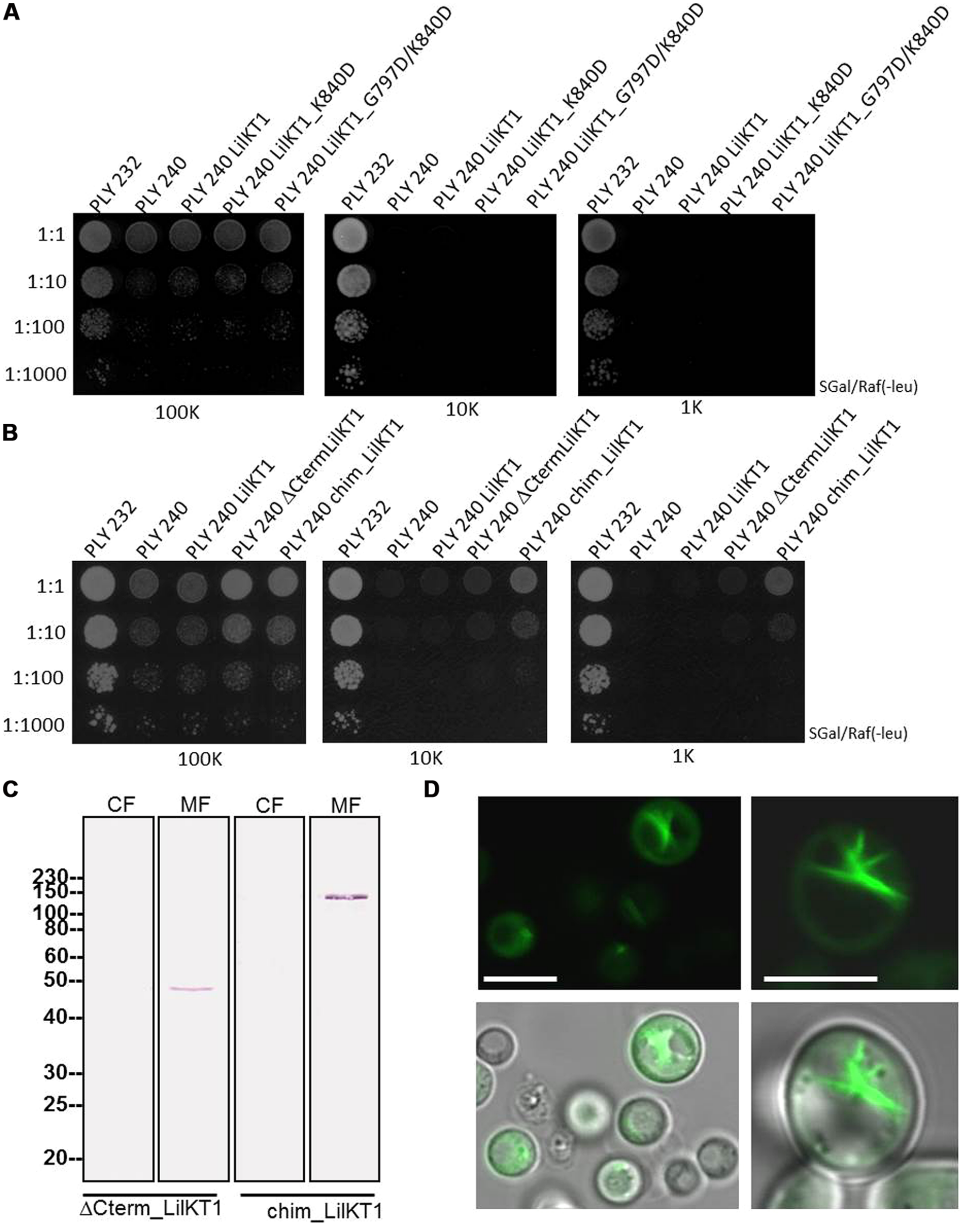
**Functional complementation of yeast mutants with modified LilKT1 proteins**. **(A)** Single (LilKT1_K840D) and double mutations (LilKT1_G797D/K840D) of the di-acidic motifs in the C-terminus of LilKT1. **(B)** Expression of LilKT1 with a total deletion of the C-terminus (ΔCtermLilKT1) and expression of chimeric protein with the N-terminus of LilKT1 and the C-terminus of AKT1 (chimLilKT1) did partially complement the growth deficiency of PLY240 in low K^+^ media. **(C)** Detection of the C-terminal deleted and chimeric LilKT1 in the cytosolic (CF) and microsomal (MF) fraction of yeast cells (PLY240) using an anti-HA-tag antibody (1:10,000) and a goat anti-mouse IgG AP-conjugated antibody (1:50,000). Predicted molecular weight s for ΔCtermLilKT1 and chimLilKT1 are 47.6 and 106.8 kDa, respectively. Channels were cloned into pGREG535. **(D)** Fluorescence localization of GFP::yLilKT1/AKT1 chimera in yeast protoplasts (left) and cells (right). Bar = 5 μm.

Recent studies (Mikosch et al., 2006) reported on the importance of C-terminal di-acidic motifs for the incorporation of K^+^ channels into the PM. Therefore, a single and a double mutant of LilKT1 were generated to incorporate additional di-acidic motifs which are present in AKT1 but not in LilKT1 (smLilKT1 G797D and dmLilKT1 G797D+K840D), the entire C-terminus of LilKT1 was replaced by the C-terminus of AKT1 (chimLilKT1) and finally, a LilKT1 mutant without a C-terminus (ΔCtermLilKT1) was generated (**Figures 1** and S4). Surprisingly, incorporation of additional di-acidic motifs could not restore the K^+^ uptake mutant and PLY240 yeast cells expressing smLilKT1 or dmLilKT1 did not grow in low K^+^ media (**Figure 6A**). In contrast to these results, the chimeric LilKT1, in which the endogenous C-terminus was replaced by the AKT1 C-terminus, and also the truncated LilKT1 without any C-terminus could complement the K^+^ uptake mutants (**Figure 6B**). The complementation assay also showed that the truncated ΔCtermLilKT1 was less effective than the chimeric LilKT1. Faint yeast growth was still visible in a 1:10 dilution at 1 mM KCl whereas no growth of yeast cells was detectable with the wild-type LilKT1 (*n* = 3). Both, the truncated and the chimeric LilKT1 were expressed in the yeast cells and could be monitored in the MF by immunodetection using anti HA-antibodies (**Figure 6C**). However, immunolocalization of GFP-tagged chimeric LilKT1 in intact yeast cells did not show any fluorescence signals from the PM but from endomembranes, spindle-like internal structures and partially from the cytosol (**Figure 6D**). Although the localization of the chimeric LilKT1 is a prerequisite for a functional rescue of the K^+^ uptake mutants, the GFP-tagged chimLilKT1 could not be detected in the PM, thus supporting the assumption that a very low number of functionally active LilKT1 proteins is sufficient for complementation but still too less for fluorescence detection.

In addition, the other pollen K^+^ channel AKT6 (= SPIK, Mouline et al., 2002) was also not able to complement the yeast uptake mutant PLY240 (Figure S5 and Herve Sentenac, University of Montpellier, personal communication). One might speculate that yeast cells are unable to direct pollen (gametophyte)-specific channels to the PM unless they exhibit the trafficking signatures of “sporophytic” channels although possible effects of the N-terminal tags (HA or GFP) cannot be ruled out.

### EXPRESSION OF LilKT1 IN LILY POLLEN

**FIGURE 7:**
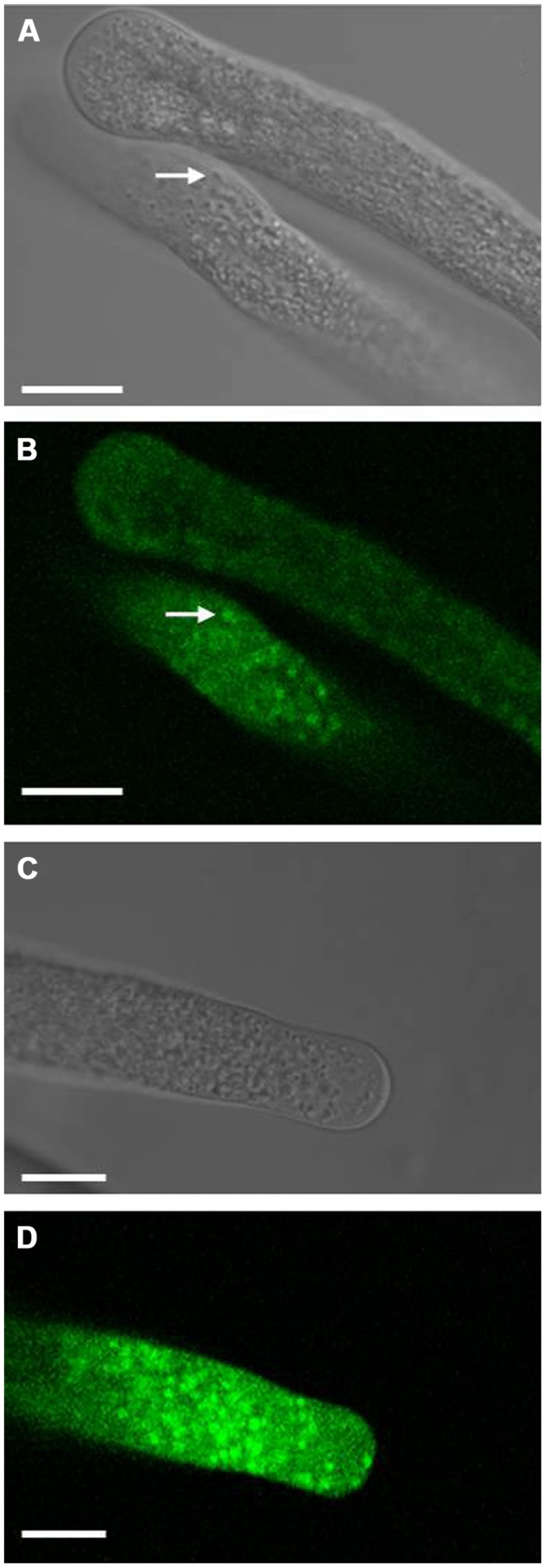
**Localization of fluorescence-tagged LilKT1 in lily pollen.** Localization of YFP fused to the C-terminus **(A,B)** or to the N-terminus **(C,D)** of LilKT1 by confocal laser-scanning microscopy. Lily pollen grains were transformed by particle bombardment with the respective plasmids, incubated for 24 h and finally transferred to germination medium. Arrows mark typical fluorescent structures inside the cytosol. Bright field images **(A,C)** of the respective confocal fluorescence images **(B,D)**. Bar = 10 μm.

To detect LilKT1 in lily pollen, pollen grains were transformed by particle bombardment to allow the expression of fluorescence-tagged LilKT1 under the control of the pollen-specific promoter LAT52 (pLAT52-LilKT1::YFP and pLAT52-YFP::LilKT1). Surprisingly, no fluorescence signals neither from the C-terminally nor from the N-terminally tagged LilKT1 were observed in the PM (**Figure 7**). Rather punctual fluorescence signals were observed in the cytosol of the pollen tube tip and in the tube shank (**Figures 7B,D**) correlating with small cytosolic particles which moved with the cytoplasmic streaming (arrows in **Figures 7A,B**). This fluorescence pattern was also observed when lily pollen was transformed with LilKT1 in the GATEWAY-compatible expression vectors p2YGW and p2GWY with LAT52 or ZM13 promoter (data not shown). No distinct fluorescence of the PM was observed. To observe an assumed early expression of LilKT1 in pollen grains without the strong autofluorescence signal of the cell wall, protoplasts were isolated from transformed, non-germinated pollen grains. Again, no consistent staining of the PM alone could be observed when compared with protoplasts expressing GFP in their cytosol (Figure S6). Although probable localization artifacts have been avoided by investigating both the N- and the C-terminally tagged LilKT1 one may still relate this unusual localization of a shaker-type channel to the overexpression of LilKT1 by the strong promoters LAT52 or ZM13, thus leading to an accumulation of LilKT1 proteins in trafficking organelles. To avoid any artificial labeling or expression, LilKT1 peptides of non-treated, native pollen tubes were identified by mass spectrometry (**Table 1**). Pollen organelle vesicles were separated by discontinuous sucrose gradient centrifugation, the proteins of each interphase separated by gel electrophoresis, gel lanes were cut into pieces and the peptides resulting from trypsin digestion were analyzed by mass spectrometry (Pertl et al., 2009). As has been shown previously (Pertl et al., 2005, 2009), the collected interphases represent enriched organelle fractions: interphase 18/25 is enriched in tonoplast, 25/30 in ER, 30/34 in Golgi membranes, 34/38 in mitochondria and interphase 38/45 is a PM-enriched fraction. Although K^+^ channels are low abundance proteins, five different peptide sequences of LilKT1 were detectable (**Figure 1A**, underlined sequences) but none of the peptides could be detected in the PM-enriched fraction (interphase 38/45). The majority of LilKT1 peptides were found in the low density fractions ranging from tonoplast to Golgi membranes (**Table 1**) thus supporting the fluorescence microscopy observations.

**Table 1:** Distribution and identification of LilKT1 peptides in a lily pollen organelle membrane preparation.

Enriched organelle fraction [sucrose interphase, % (w/w)]	No. of identified LilKT1 peptides
Tonoplast (18/25)	3
ER (25/30)	2
Golgi (30/34)	2
Mitochondria (34/38)	1
Plasma membrane (38/45)	0

*For identified peptide sequences see **Figure 1**.*

### HETEROLOGOUS EXPRESSION OF LilKT1 IN TOBACCO

**FIGURE 8:**
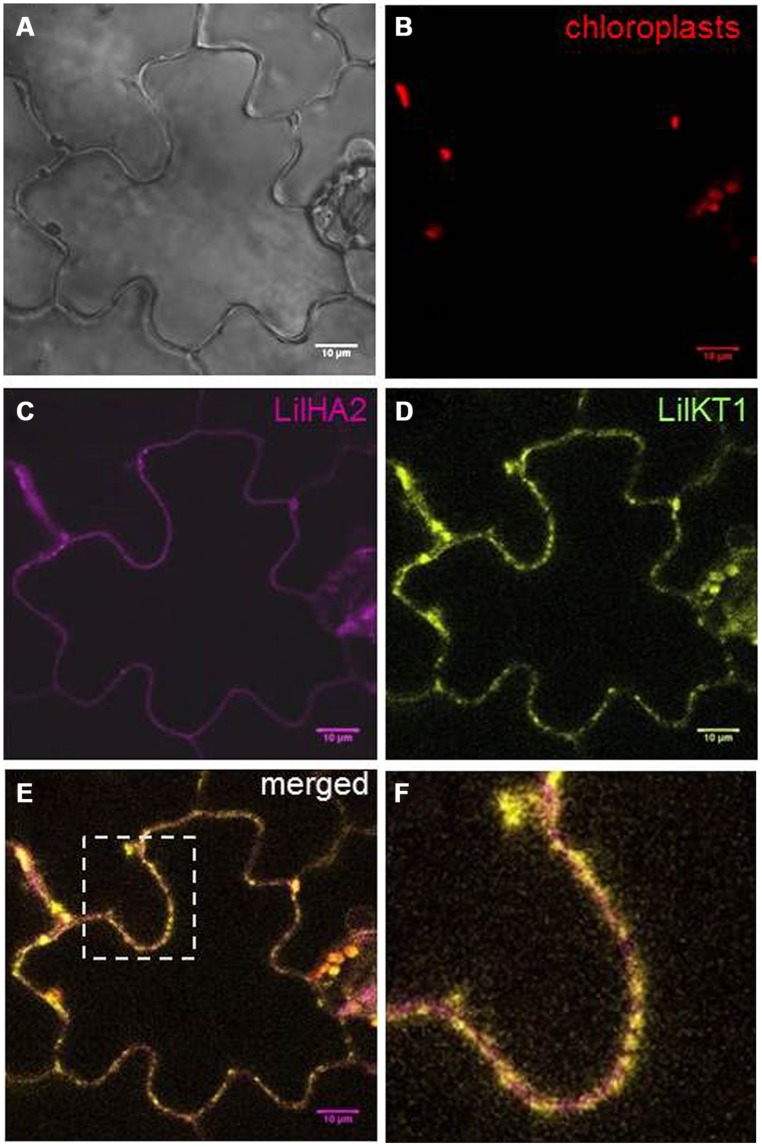
**Localization of fluorescence-tagged LilKT1 in tobacco epidermis cells**. *Nicotiana tabacum* leaves were transiently transformed by Agro-infiltration and fluorescence was monitored by confocal laser-scanning microscopy 2 days after transformation. **(A)** Bright field image. **(B)** Chloroplast autofluorescence in red. **(C)** Localization of the PM H^+^ ATPase LilHA2 fused to eCFP in magenta. **(D)** Fluorescence image of LilKT1 with N-terminal fusion of eYFP as green signals. **(E)** Merged fluorescence images of LilHA2 (magenta) and LilKT1 signals (green), overlapping signals in yellow. **(F)** Detailed view of the indicated image part of **(E)**. Bar = 10 μm.

Finally, the expression of LilKT1 was also tested in a plant expression system and YFP was fused to the N-terminus of LilKT1 by incorporation into a GATEWAY-compatible plant expression vector under the control of the CaMV 35S promoter (pK7Y-LilKT1.2, pK7C-LilHA2.2). Tobacco leaves were infiltrated with Agrobacteria containing the eYFP::LilKT1 plasmid and an eCFP::LilHA2 plasmid as control. Fluorescence was observed in tobacco leaf epidermal cells 2 days after Agro-infiltration (**Figure 8**). The fluorescence of eCFP is evenly distributed in the PM and reflects the expected localization of the PM H^+^ ATPase LilHA2 (**Figure 8C**). LilKT1 can also be observed in or very close at the PM but shows a punctuate fluorescence (**Figure 8D**). Merging both fluorescence signals clearly shows the difference in the localization patterns of the pollen PM H^+^ ATPase LilHA2 and the pollen K^+^ channel LilKT1 in the PM (**Figure 8E**), which is given in higher resolution in **Figure 8F**. Alternation of magenta and yellow signals indicated a spot-like assembly of LilKT1 in the PM in contrast to the even distribution of the PM H^+^ ATPase.

**FIGURE 9:**
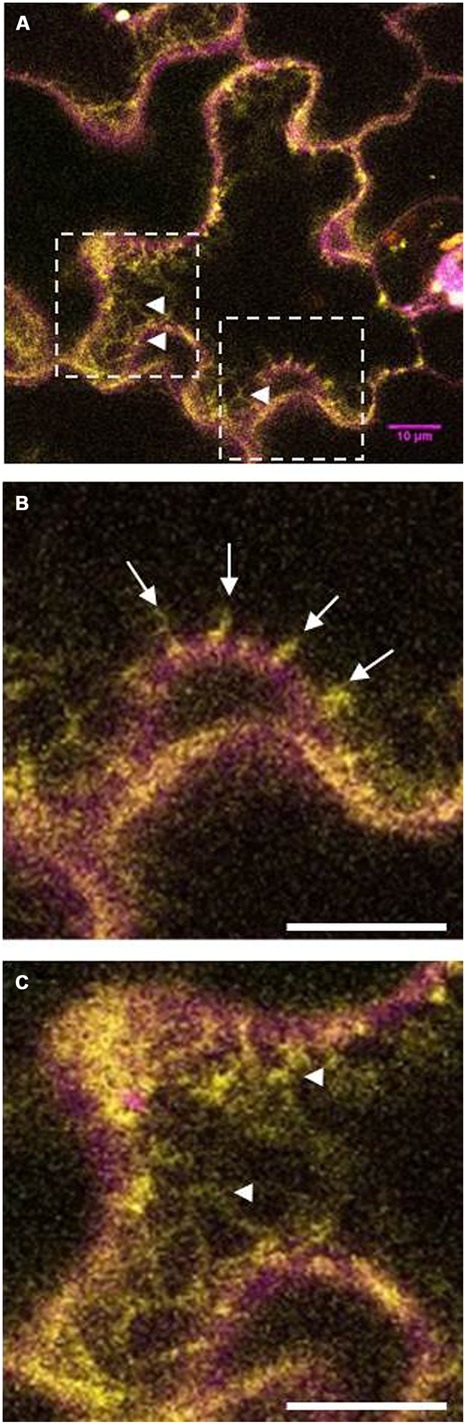
**Details of fluorescence localization of LilKT1 in tobacco epidermis cells.** Single fluorescence images of eCFP::LilHA2 (magenta) and eYFP::LilKT1 (green) were merged. Yellow signals indicate a co-localization of LilHA2 and LilKT1 in the plasma membrane. **(A)** Overview of a epidermal cell showing internal localization (green signals) of LilKT1. Boxes indicate the regions of the detailed images of **(B,C)**. **(B)** Detail showing the sub-plasma membrane localization of LilKT1 (arrows). **(C)** Localization of LilKT1 in intercellular strands (green, arrow heads) connecting LilKT1 spots (yellow) in the plasma membrane. Bar = 10 μm.

At least in tobacco leaves, LilKT1 seems to be partially directed to the PM but the majority of the epidermal cells also showed fluorescence signals originated from internally localized LilKT1 (**Figure 9**). In addition to its localization in the PM which is represented by yellow signals in the merged fluorescence images, greenish signals representing LilKT1 localization not overlapping with the PM localization of LilHA2. The greenish signals can be observed very close beneath the PM (**Figure 9B**, arrows) or in strands connecting spots of LilKT1 in the PM (**Figure 9C**, arrow heads).

## DISCUSSION

The uptake of K^+^ is essential for the pollen, which is indicated by the endogenous K^+^ inward fluxes that accompany pollen grain germination and pollen tube growth (Weisenseel and Jaffe, 1976; Messerli et al., 1999; Michard et al., 2008) as well as by the presence of large K^+^ currents across the PM mediated by voltage-gated K^+^ channels observed in *Lilium*, *Brassica,* and *Arabidopsis* pollen protoplasts (Obermeyer and Kolb, 1993; Fan et al., 1999, 2001; Griessner and Obermeyer, 2003). *Arabidopsis* mutants lacking the K^+^ inward rectifying channel exhibited reduced tube growth (Mouline et al., 2002) suggesting that a fast uptake of K^+^ might be involved in regulating the water uptake during pollen tube growth and also controls the turgor pressure (Benkert et al., 1997; Becker et al., 2004; Pertl et al., 2010; Winship et al., 2010) in analogy to the movement of stomata (Roelfsema and Hedrich, 2005). In this case, the inward and outward fluxes of K^+^ have to be tightly controlled to regulate the osmotic pressure of the cytosol to prevent bursting of the pollen tube by balancing the cytosolic osmolyte concentrations and the water uptake during the fast tube elongation. Additionally, pollen tubes may use the K^+^ uptake via channels to respond rapidly to changes in the osmotic conditions on their way through the style tissue thus, ensuring a successful fertilization. So far, only one K^+^ inward rectifying channel has been identified and characterized, AKT6 of *Arabidopsis* pollen (Mouline et al., 2002) although inward currents could be observed in all pollen species studied. The AKT1-like K^+^ channel from *Lilium* pollen identified here, resembles all characteristic features of shaker-type, plant inward rectifying K^+^ channels (Pilot et al., 2003): six transmembrane domains, a positively charged S4-transmembrane domain, the K^+^ selectivity filter GYGD, and of course a high homology to other plant K^+^ inward rectifiers (**Figure 1**). Therefore, a localization of LilKT1 in the PM was expected and the electrophysiological characterization of functional LilKT1 channels in yeast was planned. Surprisingly, LilKT1 failed to complement the K^+^ uptake yeast mutant strains WΔ3 and PLY240 (**Figures 3** and S3) and GFP-tagged LilKT1 was not detected in yeast PMs. Only co-expression with AKT1, resulted in co-sedimentation of LilKT1 together with AKT1 and the PM H^+^ ATPase, Pma1p in yeast cells (**Figure 4**). But again, no fluorescence signals were visible when GFP-tagged LilKT1 was co-expressed with AKT1 (**Figure 5**) although the previous experiment indicated a localization in the PM. Therefore, one may conclude that the amount of LilKT1 in the PM was extremely low and LilKT1 could only be detected after concentration of pooled PM fractions from three preparations (1.5 l yeast culture) and more sensitive techniques like immunodetection and chemiluminescence. Nevertheless, LilKT1 could be directed to the yeast PM when co-expressed with another K^+^ channel subunit.

Di-acidic motifs at the C-terminus have been described to be important for the escape of membrane proteins from the ER (Mikosch et al., 2006; Zelazny et al., 2007; Mikosch and Homann, 2009). However, no fluorescence signals of the GFP-tagged LilKT1 mutants were detectable at the PM when AKT1-specific diacidic motifs were introduced into the LilKT1 C-terminus. Only the chimeric LilKT1 containing the entire AKT1 C-terminus could complement the K^+^ uptake mutant PLY240. Therefore, a still not identified ER-retention signal might be located in the C-terminus of LilKT1 or a specific ER export signal is missing in LilKT1. This assumption is further supported by the result that mutant yeast (PLY240) cells co-expressing LilKT1 with AKT1 or KAT1 are growing slower than the yeast cultures expressing only AKT1 or KAT1. In addition, the amount of AKT1 in the PM (or the immunodetection signal) is slightly reduced when LilKT1 is co-expressed. It has to be noted that the other pollen K^+^ channel, AKT6, cannot complement yeast K^+^ uptake mutants either. One may therefore conclude that the somehow more tightly regulated ER-export might be a characteristic of pollen K^+^ channels although no pollen-specific sequences are observed when comparing with the amino acid sequence of AKT1 (Figure S5).

In contrast to yeast, the LilKT1 channel protein fused to YFP seems to enter the PM of tobacco epidermal leaf cells (**Figures 8 and 9**) indicating that the proposed, unknown ER-retention signal can be partially over-ruled in plants. But compared with the PM H^+^ ATPase LilHA2, LilKT1 exhibits a punctuate fluorescence in the PM similar to that reported for KAT1 (Meckel et al., 2004; Sutter et al., 2006) and could. also be observed in sub-PM and ER-like structures (**Figure 9**) where the protein is probably waiting for PM incorporation mediated by SNARE proteins (Sutter et al., 2006). In lily pollen, neither YFP-tagged nor the native LilKT1 protein were localized in the PM.

These results allow the following hypotheses: (i) LilKT1 is not a PM channel, (ii) the amounts of functional LilKT1 in the PM are below the detection limits of fluorescence microscopy and mass spectrometry analysis, or (iii) LilKT1 needs an additional factor for delivery to the PM during heterologous expression. Usually, shaker-type ion channels are voltage-gated and the K^+^ inward rectifiers open at voltages more negative than –100 mV (Griessner and Obermeyer, 2003). Where else is such hyperpolarization observed, if not in the PM of a cell? No voltage-gated, shaker-type inward rectifier was functionally detected in organelles other than the PM (Lebaudy et al., 2007; Sharma et al., 2013).

The second hypothesis can be tested with already published data. In pollen tubes of *L. longiflorum*, the peak K^+^ flux density is 283 ± 54 pmol cm^-2^s^-1^ with an extrapolated flux density of 688 ± 144 pmol cm^-2^s^-1^ at the membrane surface (Messerli et al., 1999) gives an average K^+^ flux density of 300 pmol cm^-2^s^-1^ corresponding to approximately 300 fA μm^-2^. Growing lily pollen tubes showed a resting membrane potential around –100 mV and patch-clamp experiments revealed inward single channel cation currents of 5 pA at –100 mV in lily pollen (Obermeyer and Kolb, 1993). This gives a current density *I/A*:

$$I/A=i\times P_{open}\times N/A$$

with *i* = single channel current, *P_open_* = open probability, and *N/A* channel density.

Assuming a K^+^ current density of 0.3 pA μm^-2^ in a growing pollen tube, a low open probability of 10% and a single channel current of 5 pA, thus a channel density of 0.6 μm^-2^ or 60 active channels per 100 μm^2^ would be sufficient to generate the measured currents. Because only a tenth of the K^+^ channels are active, one may expect 600 channels per 100 μm^2^ or in total 1,500 in a protoplast with a diameter of 45 μm. This is probably far too less for fluorescence detection and also for mass spectrometry. But why are not more channels incorporated into the PM when overexpressed in pollen like in the tobacco epidermis cells? We assume that for constant tube growth, the number of active K^+^ channels in the PM has to be limited and thus the incorporation of K^+^ channels is tightly regulated. Assume a large number of K^+^ channels open due to a hyperpolarizing signal: the cytosol gets flooded with K^+^ and water is following its potential gradient, the turgor increases and the tube bursts. Therefore, to prevent irregular incorporation into the PM, the pollen-specific K^+^ inward rectifiers might contain still unidentified signal sequences which enable a much tighter trafficking control than for sporophytic K^+^ channels. It may be assumed that both a PM-directing signal and a retention signal are located on the C-terminus of the channel because subdomain swapping of the entire C-terminus between LilKT1 and AKT1 and deletion of the C-terminus complemented, at least partially for the deletion, the yeast K^+^ uptake mutants. This postulated trafficking signal probably works in addition to the C-terminal di-acidic motifs and to the recently discovered amino acids V and S in the C-linker region which determined PM localization of KAT2 (Nieves-Cordones et al., 2014) and which are present in LilKT1 (**Figure 1**). Similarly to LilKT1, the *Arabidopsis* K^+^ channel SPIK/AKT6 which is exclusively expressed in pollen (Mouline et al., 2002) is also not transferred to the PM in yeast K^+^ uptake mutants.

## CONCLUSION

In summary, we postulate that pollen-specific delivery and recycling are the reasons why expression of fluorescent LilKT1 shows non-PM localization in yeast and pollen as well as partially in leaf epidermis cells, and not a special role of the pollen K^+^ inward channel in endomembranes because K^+^ currents of pollen grains and tubes showed similar characteristics as K^+^ inward currents of other tissues. All conclusions make us believe that LilKT1 is also present in the PM although much more sensitive methods are needed for detection.

## AUTHOR CONTRIBUTIONS

The entire study was planned and designed by Adam Bertl and Gerhard Obermeyer who also wrote the manuscript and analyzed the data. Full-length sequences of LilKT1 were obtained by Peter Lughofer and Rene Hude. Transformation, analysis, and localization of LilKT1 in yeast were performed by Minou J. Safiarian, Peter Lughofer, Adam Bertl, and Heidi Pertl-Obermeyer. Mass spectrometry analysis was performed by Heidi Pertl-Obermeyer, plant transformation and fluorescence imaging experiments were conducted by Minou J. Safiarian (tobacco and pollen) and by Peter Lughofer (pollen).

## Conflict of Interest Statement

The authors declare that the research was conducted in the absence of any commercial or financial relationships that could be construed as a potential conflict of interest.

## Footnotes

1 http://gateway.psb.ugent.be

2 http://openwetware.org/wiki
