# Supplementary material for: Lost in traffic? The K+ channel of lily pollen, LilKT1, is detected at the endomembranes inside yeast cells, tobacco leaves, and lily pollen
Source: Front Plant Sci. 2015 Feb 10;6:47. doi: 10.3389/fpls.2015.00047 (PMC4322604; doi:10.3389/fpls.2015.00047)
Supplement: Supplementary file 1 [file Data_Sheet_1.PDF]

Tab. S1 Sequences of primer pairs used for cloning and verification of the cloned inserts, e.g. M13 primer pairs. Inosin was used in degenerated primer sequences instead of all nucleotides (i = inosin).

**primer name**	**sequence from 5' → 3'**
H5_fwd	aa(ag) ac(i) tgg at(act) gg(i) gc
S6_rev	ac(i) ag(ag) tti gtc at(ag) tti cc
S6spec_rev	acg agg ttg gtc atg ttg cc
H5spec_fwd	aaa acg tgg atc ggg gc
H5spec_rev	gcc ccg atc cag gtc tt
GYGDHLA_rev	tgc atg caa gtc gcc ata tcc
M13_fwd	gta aaa cga cgg cca g
M13_rev	cag gaa aca gct atg ac
LilKT1_fwd	atg tgc ggc cac gag cc
LilKT1_rev	tta aga ata ggc ctg act tcc cct c
Rec1_AKT6_fwd	gat acc gtc gac aat gga gaa gaa g
Rec2_AKT6_rev	cga ggt cga ctc aag gat ccc
**Primer pairs for construction LilKT1/AKT1 chimera by SOE-PCR**	
SOE_N_LilKT1_rec1_fwd	gaa ttc gat atc aag ctt atc gat acc gtc g
SOE_N_LilKT1_rev	cat ttg atc ttg caa tct aac tgg
SOE_AKT1_C_fwd	cca gtt aga ttg caa gat caa atg ctt gca c
SOE_AKT1_C_rev	atg act cga ggt cga ctc aag aat cag ttg caa aga tg
**Primer pairs for site-directed mutagenesis by Around-The-Horn method**	
G797D_fwd	ata atg ccc cag caa aat tgg tat tgt tgc c
G797D_rev	ctt ttt cag gac atc taa tag tga ctc ttg gtg gtg
K840D_fwd	gac ttg gtt aga gat ggt gat tgt ttg ttg
K840D_rev	aac gtc atc gat ctc tgc tcc atc tct tg

Tab. S2: Proteins with highest homologies to LilKT1. The amino acid sequence of LilKT1 was used for a BLAST search in the non-redunadant NCBI data base.

name	Max score	Total score	Query cover	E value	Max identity	Access no
LilKT1 [*Lilium longiflorum*]	1784	1784	100%	0.0	100%	ABO15470.1
putative K^+ channel AKT1, [*Ricinus communis*]	1240	1240	99%	0.0	69%	XP_002529373.1
Predicted K^+ channel AKT1-like [*Vitis vinifera*]	1232	1232	98%	0.0	69%	XP_002281787.1
unnamed protein product [*Vitis vinifera*]	1229	1229	98%	0.0	69%	CBI28150.3
Predicted K^+ channel AKT1-like [*Cucumis sativus*]	1227	1227	98%	0.0	69%	XP_004149890.1
inward rectifying shaker-like K^+ channel [*Vitis vinifera*]	1220	1220	98%	0.0	69%	CAZ64538.1
shaker-like K^+ channel 1 [*Populus euphratica*]	1216	1216	98%	0.0	69%	ADA79674.1
K^+ uptake channel [*Zea mays*]	1209	1209	98%	0.0	69%	CAI77627.1
hypothetical protein 03g029520 [*Sorghum bicolor*]	1207	1207	98%	0.0	68%	XP_002458234.1
K^+ channel [*Solanum tuberosum*]	1204	1204	99%	0.0	69%	CAA60016.1
AKT1-like K^+ [*Triticum aestivum*]	1203	1203	98%	0.0	68%	AAF36832.1
inwardly rectifying K^+ channel AKT1 [*Hordeum vulgare*]	1200	1200	98%	0.0	68%	ABE99810.1
inwardly rectifying K^+ channel subunit [*Daucus carota*]	1200	1200	98%	0.0	69%	CAG27094.1
OsAKT1 (Oryza sativa)	1199	1199	98%	0.0	66%	P0C550.1
K^+ channel [*Solanum lycopersicum*]	1199	1199	99%	0.0	68%	NP_001234258.1
OsAKT1, Os01g0648000 [*Oryza sativa*]	1198	1198	98%	0.0	66%	NP_001043713.1

```
LilKT1 -------------------------MCGHEPAE-QEMSRDG--SHYSLSSGILPSLGA--
AKT1   -------------------MRGGALLCGQVQDEIEQLSRES--SHFSLSTGILPSLGA--
AKT6   MEKKKVWFWGVKDDGEGGGGRGGGRTKDAEDDVADHLSRDGTMSQYSLSKGLLPSLGANN

                          __________S1___________         _______S2
LilKT1 RSHRKAKLRWFIISPYDQRYRIWETFLVLLVIYTAWVSPFEFGFMEHAIGALAIADNIVN
AKT1   RSNRRVKLRRFVVSPYDHKYRIWEAFLVVLVVYTAWVSPFEFGFLRKPRPPLSITDNIVN
AKT6   RSSRDVILPRFIVSPFDPRYRAWETFLVFLVLYTAWASPFEFGFLQKPRPPLSILDNIVN

       S2__                                _________S3__________
LilKT1 GLFFIDIVLTFFVAYLDKTTYLLIDSPKEIAWKYTTSWFILDIVSTIPSELARQLLPPKL
AKT1   AFFAIDIIMTFFVGYLDKSTYLIVDDRKQIAFKYLRSWFLLDLVSTIPSEAAMRI---SS
AKT6   GFFAVDIVLTFFVAFLDKVTYLLVDDPKRIAWRYASTWLIFDVVSTFPYEIFGSLLHESI

                _________S4___________     _________S5__________
LilKT1 RSYGFLNMLRLWRLRRVSCLFARLEKDRNFNYFWVRCAKLIFVTLFAVHCAGCFYYLIAA
AKT1   QSYGLFNMLRLWRLRRVGALFARLEKDRNFNYFWVRCAKLVCVTLFAVHCAACFYYLIAA
AKT6   QGYGIFSMLRLWRLRRVSNCFARLEKDRKYSYFWVRCSKLLLVTLFVIHCGACFLYSIAA

                                                             ______
LilKT1 RYHDPTKTWIGASMPDFHEQSLWVRYVTSMYWSITTLTTVGYGDLHAQNTGEMIFDIAYM
AKT1   RNSNPAKTWIGANVANFLEESLWMRYVTSMYWSITTLTTVGYGDLHPVNTKEMIFDIFYM
AKT6   HYPDPSKTFMALTDENWKESPIAVRYNTAMYWSITTFSTTGYGDIHGVNSREMTFILFYM

       ______S6________
LilKT1 LFDLGLTAYLIGNMTNLVVHCTSRTRKFRDTIQAASSFALRNQLPVRLQDQMVAHLCLKF
AKT1   LFNLGLTAYLIGNMTNLVVHGTSRTRNFRDTIQAASNFAHRNHLPPRLQDQMLAHLCLKY
AKT6   VFNLGLSAYIIGNMTNLVVHVTGRTRKFRDTIQAASGFGQRNNLPVRLQDQMVAHLCLRY

LilKT1 RTDSEGLQQQETLDALPKAIRSSISHYLFYTLVNKVYLFRGVSHDLLFQLVSEKKAEYFP
AKT1   RTDSEGLQQQETLDALPKAIRSSISHFLFYSLMDKVYLFRGVSNDLLFQLVSEMKAEYFP
AKT6   RTDSEGLQQQEIIDSLPKAIRSSISHYLFYEVVDKIYLFHGISNDLLFQLVTEMKAEYFP

LilKT1 PREDVILQNEAPTDFYILVTGSVDLVDHKNGIEQIVREANPGELVGEIGVLCYRPQLFTI
AKT1   PKEDVILQNEAPTDFYILVNGTADLVDVDTGTESIVREVKAGDIIGEIGVLCYRPQLFTV
AKT6   PKEDVILQNEAPTDFYILVTGAVDIIARVNGVEQVVSEAQRGHVFGEVGVLCYRPQLFTV

LilKT1 RTKKLCQLLRLNRNSFLSIVQSNVGDGTVTMNNLLQYLKEQKDHVMQGVLRETGNMLARG
AKT1   RTKRLCQLLRMNRTTFLNIIQANVGDGTIIMNNLLQHLKEMNDPVMTNVLLEIENMLARG
AKT6   RTKRLSQLLRLNRTVLLNLVQANVGDGAIIMNNLLQHLKDSEDPVMKGVLADTEHMLAQG

LilKT1 RLDLPLTLCFAATRGDDLLLHQLLRRGLDPNESDNNGWSALHIAASKGNESCVVLLLDFG
AKT1   KMDLPLNLCFAAIREDDLLLHQLLKRGLDPNESDNNGRTPLHIAASKGTLNCVLLLLEYH
AKT6   KMDLPLSLCFAAARGDDLLLHQLLRRGSSPNEMDKDGRTALHIAASKGSHYCVVLLLEHG

LilKT1 ADPNCRDSEGRVPLLEAILGKHDSVVRVLVDHGADLSSGDAAQYACIAAEQNNLELLQSI
AKT1   ADPNCRDAEGSVPLWEAMVEGHEKVVKVLLEHGSTIDAGDVGHFACTAAEQGNLKLLKEI
AKT6   ADPNIRDSEGNVPLWEAIIGRHREIAKLLAENGAKLSLDSVSYFSGLAVEKNCLDALKDI

LilKT1 VQYGGDISAPKLDGNTALHIAVTEGNVPIVKFLLEHGAEIDKPDSHGWTPRGLADQQSHE
AKT1   VLHGGDVTRPRATGTSALHTAVCEENIEMVKYLLEQGADVNKQDMHGWTPRDLAEQQGHE
AKT6   IKYGGDVTLPDGNGTTALHRAVSEGHLEIVKFLLDQGADLDWPDSYGWTPRGLADHQGNE

LilKT1 EIKALFEAKRDIPKVSDT--------TPTSHLLGRYSSEPMIQRLSSDGILVADDNKQRR
AKT1   DIKALFREKLHERRVHIETSSSVPILKTGIRFLGRFTSEPNIRPASREVSFRIRETRARR
AKT6   EIKTLFHNHRPVEKKPKPI-PGIPQSPVTGKPLMKYSSEPTMHSGELVLDGGQVVVSQKR

LilKT1 RANNFRNSLFGIMSAAKVDREYGPLPSPSGPSRFMAVAPHHRTPPRVTIRCPEKGNAPAK
AKT1   KTNNFDNSLFGILANQSVPKNGLA----------TVDEGRTGNPVRVTISCAEKDDIAGK
AKT6   KLNNFRNSLFGIISAANSADDGGEVPRSPAV---PGGGGSMIYPERVTISSPENGETGGK

LilKT1 LVLLPGSLKELLDLGGKKFGLVLVKVLTRD-GAEIDDVKLVRDGDCLLLVSDRWRGSQAYs
AKT1   LVLLPGSFKELLELGSNKFGIVATKVMNKDNNAEIDDVDVIRDGDHLIFATDS--------
AKT6   VVLLPNSMEELLKIGENKMGFVPTKVLTRE-GAEIDDITLIRDGDFLLLSRDP--------
```

Fig.S1 Sequence alignment of LilKT1, AKT1 and AKT6. The transmembrane domains S1 – S6 are indicated. The amino acid sequence LQDQ was used as an overlapping sequence to construct the N-terminal-LilKT1 + C-terminal-AKT1 chimera. Areas with similar amino acids of all three proteins are marked in red, yellow were two sequences show amino acid similarities.

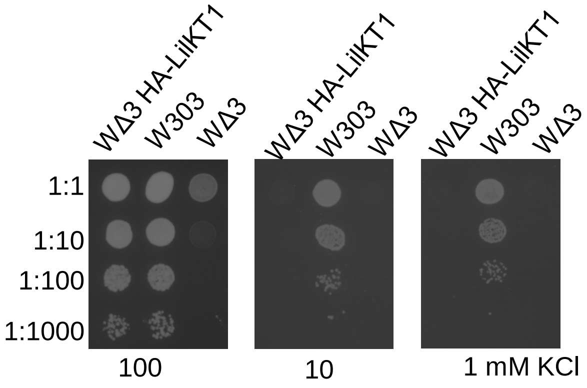

Fig. S2 Functional complementation of yeast K^+ uptake mutant WD3. Yeast mutant WD3 expressing HA-tagged yLilKT1 (HA-LilKT1) did not grow at low K^+ concentrations (1 mM KCl). Yeast cells were complemented with yLilKT1 in pGREG536 containing *URA3* as a selectable marker.

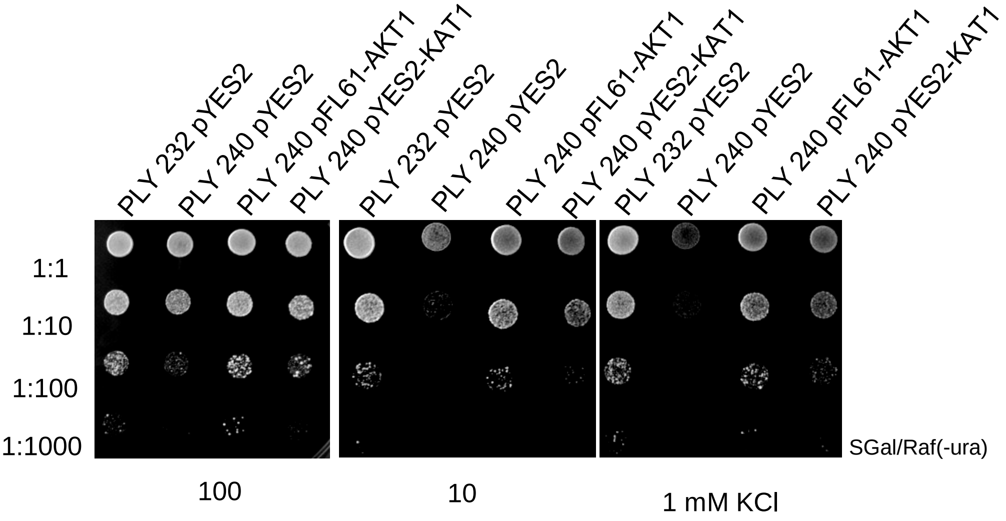

Fig. S3 Functional complementation of yeast K^+ uptake mutant PLY240. Yeast mutant PLY240 expressing the Arabidopsis K^+ channels AKT1 and KAT1 is growing in low K^+ medium (1 mM KCl).

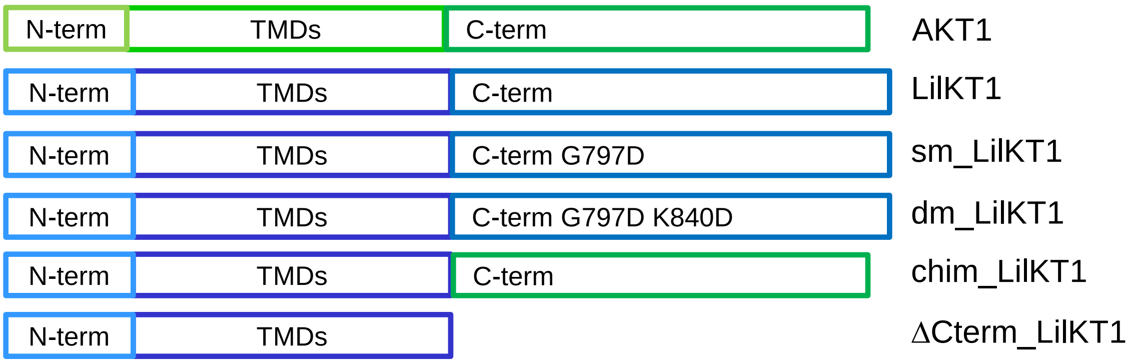


```
AKT1....PVRVTISCAE KDDIAGKLVL LPGSFKELLE LGSNKFGIVA TKVMNKDNNA EIDDVDVIRD GDHLIFATDS
LilKT1..PPRVTIRCPE KGNAPAKLVL LPGSLKELLD LGGKKFGLVL VKVLTRDG.A EIDDVKLVRD GDCLLLVSDR WRGSQAYS
       787       G797D                                                K840D
```

Fig. S4 Mutations of the amino acid sequence of the C-terminus of LilKT1. Arabidopsis AKT1 and the lily pollen channel are marked in green and blue, respectively. The C-terminal amino acid sequence alignment of both channel starts with LilKT1 proline 787. The di-acidic motifs (red) which were mutated, are underlined. sm: single mutation, dm: double mutation.

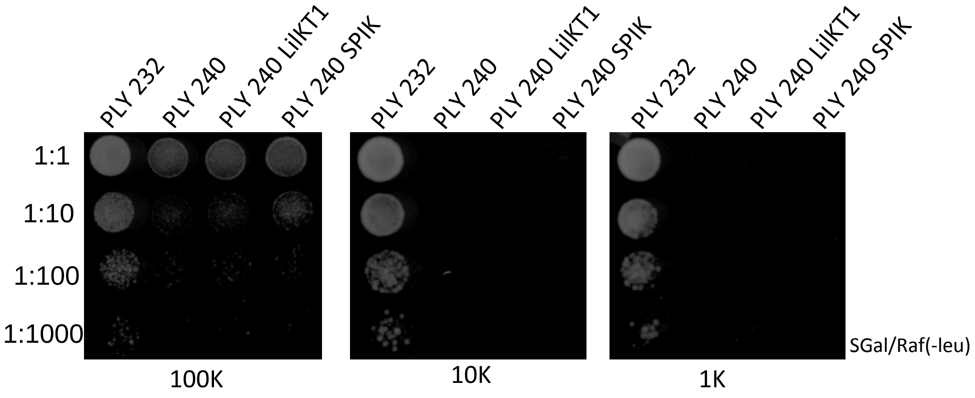

Fig. S5 Functional complementation assay of K^+ uptake mutant PLY240 with AKT6 (SPIK). Channels were cloned into pGREG535.

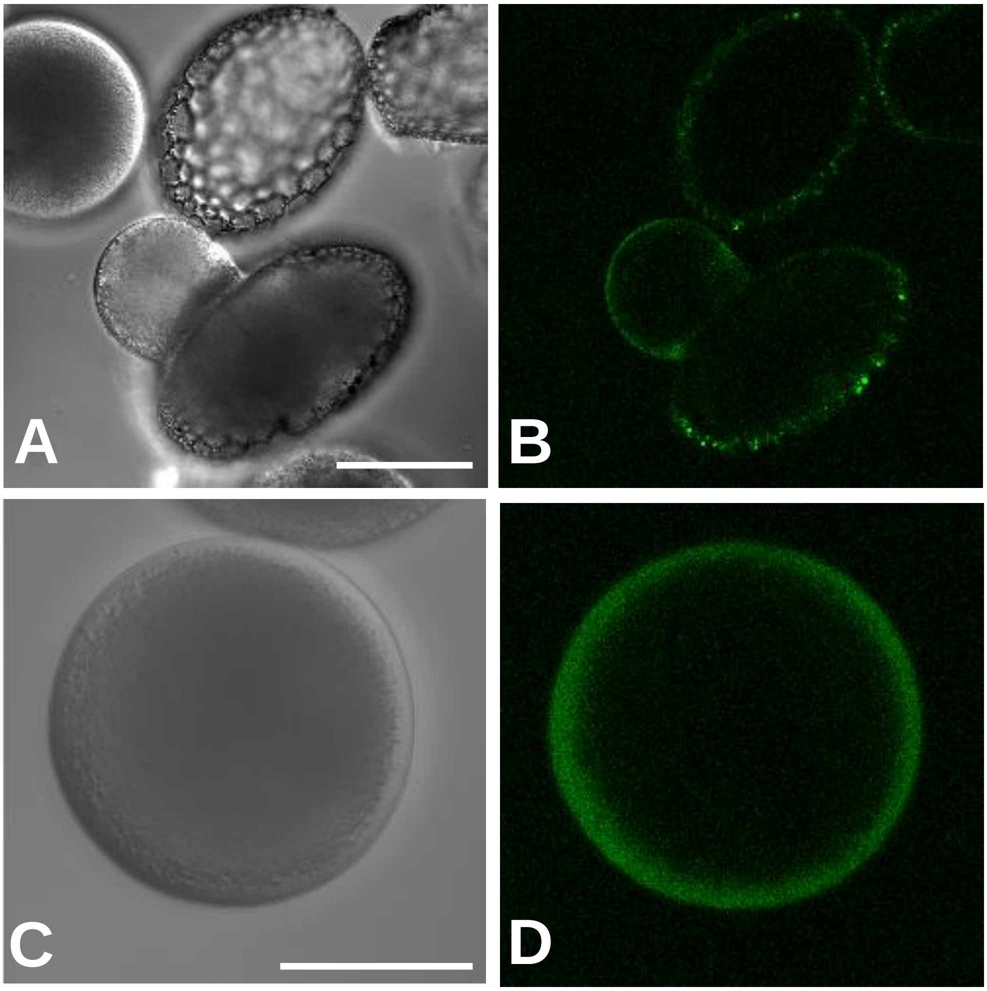

Fig. S6 Localization of LilKT1 in protoplasting lily pollen grains. (A) Bright field image of a protoplast released from a lily pollen grain and **(B)** the corresponding fluorescence image showing the fluorescence of YFP fused to the N-terminus of LilKT1. **(C)** and **(D)** Bright field and fluorescence image, respectively, of cytosolic GFP expressed in lily pollen protoplasts. Transient expression of fluorescent proteins by particle bombardment with respective plasmids. Bar = 50 µm.
